# Evolutionary history and divergence times of Tettigoniidae (Orthoptera) inferred from mitochondrial phylogenomics

**DOI:** 10.3389/fgene.2025.1495754

**Published:** 2025-03-13

**Authors:** Tianyou Zhao, Zhenbin Lin, Hailin Yang, Fan Song, Zhenyuan Xia, Weidong Huang

**Affiliations:** ^1^ Department of Entomology and MOA Key Lab of Pest Monitoring and Green Management College of Plant Protection, China Agricultural University, Beijing, China; ^2^ Yunnan Tobacco Company, Yuxi, China; ^3^ Yunnan Academy of Tobacco Agricultural Sciences, Kunming, China

**Keywords:** Tettigoniidae, mitochondrial genome, divergence time, selective pressures, evolutionary rate, phylogeny

## Abstract

**Background:**

Advances in high-throughput sequencing technology have led to a rapid increase in the number of sequenced mitochondrial genomes (mitogenomes), ensuring the emergence of mitochondrial phylogenomics, as a powerful tool for understanding the evolutionary history of various animal groups.

**Methods:**

In this study, we utilized high-throughput sequencing technology to assemble and annotate the mitogenomes of *Letana rubescens* (Stål) and *Isopsera denticulata* Ebner. We described the characteristics of the mitochondrial genes of these two species. Utilizing 13 PCGs and 2 rRNA genes, we reconstructed the phylogenetic relationships of Tettigoniidae by combining published data with our newly generated data. We used likelihood mapping, signal-to-noise ratio (SNR), and saturation analysis across different datasets to ensure the robustness of our inferred topologies.

**Results and conclusion:**

Selective pressure analysis on the 13 protein-coding genes (PCGs) and 2 ribosomal RNA (rRNA) genes revealed that only *ND1* and *COX1* contained positively selected sites, while negative selection dominated across all genes, indicating that mitochondrial genes primarily function to maintain genetic integrity. Additionally, we assessed the evolutionary rates of the 13 PCGs and two rRNA genes across five major subfamilies using mean pairwise identity analysis. Phylogenetic results of our study provide more precise insights into the relationships within Tettigoniidae, spanning subfamilies, tribes, genera, and species. We further estimated the divergence times of Tettigoniidae using four fossil calibration nodes in MCMCTree, dating the origin of katydids to the early Paleogene period (approximately 60.86 Mya), and identifying the divergence nodes for five major subfamilies.

## 1 Introduction

Tettigoniidae is a major family of “long-horned grasshoppers” in Ensifera of Orthoptera, with more than 8,300 species in 1,300 genera (Cigliano et al., 2024). They are found on all continents except Antarctica. Tettigoniids are characterized by their robust hind limbs that allow them to leap, powerful mouthparts that are adept at chewing, four distinct tarsal segments, elongated and filiform antennae (consisting of over 30 segments, sometimes surpassing their own body length), and specialized forewings that can produce sounds through stridulation (Naskrecki, 2013). The unique leaf-like wings of katydids have attracted the attention of many researchers (Nel et al., 2008; Garrouste et al., 2016; Baker et al., 2017). It has been discovered that this characteristic has independently evolved in at least six different lineages (Mugleston et al., 2013). The majority of species of Tettigoniidae are found within five large, cosmopolitan subfamilies (Conocephalinae, Tettigoniinae, Phaneropterinae, Pseudophyllinae, and Meconematinae).

Although the evolution of Orthoptera has been explored in multiple studies (Song et al., 2020; Gaugel et al., 2023), a clear assessment of the impact of mitochondrial data on phylogeny is still lacking. Mitochondrial DNA is a compact genome, typically 14–20 kb in length, that has served as a widely used genetic locus for a series of animal evolutionary studies (Cameron, 2014; Huang et al., 2023). However, the results of phylogenetic tree construction using mitochondrial data can be influenced by various factors, such as saturation (Allio et al., 2017) and site heterogeneity (Li et al., 2017; Liu et al., 2018). In addition, exploring additional mitochondrial attributes such as mutation rates and adaptive potential is also vital to fully understanding the complexities of mitochondrial evolution. For example, a mitogenomic divergence of the AT content was generally lower in Ensifera than in Caelifera (Sheffield et al., 2010; Liu et al., 2013). However, with larger samples, it has been demonstrated that there is no significant difference in the AT content between Ensifera and Caelifera (Gaugel et al., 2023). Therefore, utilizing the continuously growing database to obtain statistics on AT content helps us to gain a deeper understanding of the actual changes. Compared to the mitochondrial genes of flightless grasshoppers, those of flying grasshoppers were positively selected in response to the energy requirements of flight (Li et al., 2018). Selective pressure is a significant driving force in mitochondrial evolution (Meiklejohn et al., 2007; James et al., 2016). Through positive and purifying selection, mitochondria can continuously adapt to environmental changes, optimizing their genetic characteristics and functions.

In this study, we sequenced and annotated two complete mitochondrial genomes (mitogenomes) of Tettigoniidae species, *Letana rubescens* (Stål) and *Isopsera denticulata* Ebner. Furthermore, we inferred the phylogenetic relationships among Tettigoniidae by incorporating our two newly sequenced mitogenomes and other mitogenomes downloaded from the public database. Phylogenetic signals at key nodes were also analyzed using likelihood mapping. Understanding the proportion of sites under positive selective pressure is particularly important for exploring the evolution of the mitochondrial genes in Tettigoniidae. In order to explore the evolutionary history of mitochondria, we measured differences in selective pressure among mitochondrial genes, as well as a mean pairwise identity among the five main subfamilies. Finally, divergence times among the main clades of Tettigoniidae were estimated. Our study provides a comprehensive perspective on mitogenomic evolution among Tettigoniidae species.

## 2 Materials and methods

### 2.1 Taxon sampling and DNA extraction

Two species of Tettigoniidae, namely, *L*. *rubescens* and *I*. *denticulata*, were collected from Chengguan Town, Shibing County, Guizhou Province, China (108.163003 °E and 27.084417 °N; 767.0 m) on 14 July 2021. Fresh specimens for sequencing were collected and preserved in absolute ethanol and stored at −80°C. We pretreated the specimens with 0.9% NaCl buffer before DNA extraction, as suggested by Huang et al. (2019). Total genomic DNA was extracted from cephalothorax tissues using a DNeasy Blood and Tissue kit (QIAGEN, Valencia, CA, United States) according to the manufacturer’s instructions. The vouchers and DNA of the specimens were deposited in the Entomological Museum of China Agricultural University (Beijing, China).

### 2.2 Mitochondrial genome assembly and annotation

All Illumina TruSeq libraries were prepared with an average insert size of 350 bp and sequenced using the Illumina NovaSeq 6000 platform (Berry Genomics, Beijing, China) with 150-bp paired-end reads. Mitochondrial sequences were assembled using GetOrganelle v1.7.2a (Jin et al., 2020). Protein-coding genes (PCGs), ribosomal RNAs (rRNAs), and transfer RNAs (tRNAs) of all mitochondrial genes were uniformly annotated using MitoZ v3.3 (Meng et al., 2019). A graphical map of the annotated circular mitogenome was generated using the OGDRAW tool 1.3.1 (Greiner et al., 2019).

### 2.3 Sequence alignment and dataset selection

The amino acid sequences of PCGs and two rRNA genes were aligned using the default strategy in MAFFT v7.310 (Katoh and Standley, 2013). The nucleotide sequences of each PCG based on the aligned amino acid sequence in the previous step, were separately aligned using TranslatorX v1.1 (Abascal et al., 2010). Ambiguous sites and poorly aligned positions were pruned using ClipKIT v2.3.0 (Steenwyk et al., 2020) with a smart-gap mode. The aligned and pruned sequences were concatenated into a matrix using PhyloSuite v1.2.3 (Zhang et al., 2020). The relative synonymous codon usage (RSCU) was calculated using the CAI module (Lee, 2018). We constructed three datasets for further phylogenetic analyses. The first dataset was composed of all PCG sequences and rRNA gene sequences (P123R dataset; 13,971 bp). The second dataset was composed of all PCG sequences with the third codon position excluded and rRNA gene sequences (P12R dataset; 10,182 bp). The amino acid sequence of PCGs that excluded the termination codon was used in the third dataset as the AA dataset (3,789 AA).

### 2.4 Phylogeny reconstruction

The in-group included 93 species of Tettigonioidea, as depicted in Supplementary Table S5. The mitogenomes of two species of Hagloidea, six species of Rhaphidophoroidea, and nine species of Stenopelmatoidea were selected as out-groups. Additionally, the mitogenome of *Schizodactylus jimo* (Schizodactylidae) was downloaded from NCBI and chosen as a rooted out-group (Gaugel et al., 2023). Phylogenetic analyses were performed based on three datasets using maximum likelihood (ML) inference and Bayesian inference (BI).

ML inference was conducted in IQTREE v2.1.2 (Minh et al., 2020). Substitution models were compared and selected according to the Bayesian information criterion (BIC) by using ModelFinder (Kalyaanamoorthy et al., 2017). An edge-unlinked model was specified for both the full-partition and merged-partition schemes. A total of 1,000 ultrafast bootstraps were used to evaluate the nodal support of the ML tree (Hoang et al., 2018). For each matrix, three partition schemes were applied for ML inference: (1) no partition (NP); (2) full partition (FP), which provides the best-fitting model for each individual gene; and (3) merged partition (MP), which implements a greedy strategy starting with the full partition model and subsequently merging pairs of genes until the model fit does not improve any further. We selected the best trees according to the BIC, as depicted in Table 1.

**TABLE 1:** Detailed information on the alignment and corresponding optimal tree for three datasets. In partition scheme (t), the number in parentheses indicates the number of partitions.

Dataset	Partition scheme (t)	ln (Lik)	AIC	BIC	SNR	Saturation	BS ≥ 95	BS < 95
AA	MP(4)	−184,039.429	368,736.858	370,790.031	9.8476	0.9277	91	17
AA	FP(13)	−184,309.279	369,454.559	372,063.150	9.8111	0.9267	87	21
AA	NP(1)	−185,702.129	371,880.259	373,365.534	9.8293	0.9270	91	17
P12R	MP(7)	−622,468.835	1,245,655.671	1,248,364.232	14.0337	0.8468	82	26
P12R	FP(15)	−622,217.476	1,245,460.952	1,249,331.403	14.0147	0.8463	86	22
P12R	NP(1)	−638,497.190	1,277,474.380	1,279,285.118	13.7545	0.8604	81	27
P123R	MP(7)	−642,481.179	1,285,730.359	1,288,628.252	7.7547	0.8758	85	23
P123R	FP(15)	−642,292.872	1,285,641.745	1,289,626.349	7.7561	0.8753	88	20
P123R	NP(1)	−659,158.392	1,318,820.785	1,320,722.528	7.5811	0.8609	89	19

BI was implemented under the CAT + GTR model using PhyloBayes-MPI v1.8 (Lartillot and Philippe, 2004; Lartillot et al., 2013). Two independent Markov chain Monte Carlo (MCMC) runs of 5,000 generations each were executed. Convergence was evaluated using the “bpcomp” and “tracecomp” procedures in the PhyloBayes package with a burn-in of the first 20% by the recommended criterion of maximum discrepancy <0.3. A consensus tree was simultaneously built by pooling the remaining MCMC trees from both runs, as shown in Supplementary Figure 10.

Saturation and signal-to-noise ratio (SNR) were computed using the Phylogenomics Toolkit v1.20.0 (Steenwyk et al., 2021). The saturation value is the ratio of the true number of substitutions in a sequence to the observed number of substitutions. The closer the value of R square is to 1, the lower the saturation of the dataset. The signal-to-noise ratio refers to the ratio between the phylogenetic signal (used to derive the signal of the “real” evolutionary tree) and the data noise (which may affect the signal derived from the “real” evolutionary tree, such as heterogeneity). Thus, a higher SNR indicates that the dataset is less likely to be affected by compositional bias. The phylogenetic trees were visualized in iTOL v 6.8.1 (Letunic and Bork, 2021).

### 2.5 Calculation of selective pressure and mean pairwise identity

Nonsynonymous (dN) and synonymous (dS) substitution rates were used to infer purifying selection (dN/dS < 1) and positive selection (dN/dS > 1). Single-likelihood ancestor counting (SLAC) employs a combination of ML and counting methods to infer dN and dS substitution rates on a per-site basis for a given coding alignment and corresponding phylogeny. Selective pressures on sites within genes were calculated using the SLAC method in Hyphy v2.5.62 (Kosakovsky et al., 2020), with the topology based on the optimal tree of the AA dataset.

We conducted a selective pressure analysis on each site to determine the probability of it being under positive or negative selective pressure. Specifically, a site is classified as under positive selective pressure if the p-value of positive selection is less than 0.05. Conversely, if the p-value of negative selection is less than 0.05, the site is categorized as being under negative selective pressure. Sites with a p-value of 0.05 or higher are regarded as not experiencing significant selective pressure and are, therefore, grouped into the “other” category.

Pairwise identities can be used as a proxy for the rate of evolution of sequences. Pairwise identity is defined as the number of identical sites (including gaps) between two aligned sequences divided by the length of the alignment. Values can range from 0 (no similarity; high diversity at the given site) to 1 (perfect match; no diversity at the given site, indicating slow evolution). Mean pairwise identities were computed using the Phylogenomics Toolkit. The evolutionary rate of sequences can be inferred by analyzing the pairwise identities (Chen et al., 2017).

### 2.6 Hypothesis testing with four-cluster likelihood mapping

The information content of the datasets was evaluated using quartet-likelihood mapping in IQTREE v2.1.2 to assess the informative resolution of three datasets. This method allowed us to visualize the tree-likeness of all quartets in a single graph and, therefore, provided a robust interpretation of the phylogenetic content of a dataset. The four-cluster likelihood mapping (FcLM) method was used to assess critical nodes with respect to Tettigoniidae.

For all FcLM analyses, the following topologies and groups were defined:

T1: (G1, G2)–(G3, G4); T2: (G1, G3)–(G2, G4); T3: (G1, G4)–(G2, G3).

Hypothesis 1 Rhaphidophoridae is the sister group to Tettigoniidae.

Groups: G1: a clade including Anostostomatidae, Stenopelmatidae, and Prophalangopsidae; G2: Rhaphidophoridae; G3: Gryllacrididae; G4: Tettigoniidae.

Hypothesis 2 Sister-group relationship between Prophalangopsidae and Rhaphidophoridae.

Groups: G1: Anostostomatidae; G2: Prophalangopsidae; G3: Rhaphidophoridae; G4: Stenopelmatidae.

Hypothesis 3 Sister-group relationship between Mecopodinae and Phaneropterinae.

Groups: G1: Anostostomatidae and Stenopelmatidae; G2: Prophalangopsidae; G3: Rhaphidophoridae; G4: Gryllacrididae.

Hypothesis 4 Aeshnidae is the sister group to Cordulegastridae.

Groups: G1: Macropodidae; G2: Phaneropterinae; G3: Pseudophyllinae; G4: Other species of Tettigoniidae.

### 2.7 Divergence time estimation

Based on the phylogenetic tree obtained in this study, we employed four fossil calibration nodes to estimate the divergence time of Tettigoniidae (Supplementary Table S6). The upper- and lower-bound (Ma) values of fossil records were uniformly provided by the Paleobiology Database (https://paleobiodb.org/). The AA dataset and its corresponding optimal consensus tree were used for subsequent analysis.

Divergence time estimation was performed using MCMCTree in PAML 4.9j (Yang, 2007), which performs Bayesian estimation of species divergence times using soft fossil constraints with the molecular clock under the auto-correlated rates model. The Dirichlet-gamma prior to the overall substitution rate (rgene gamma) was set to G (1, 11.03), calculated by baseml. The Dirichlet-gamma prior to the rate-drift parameter (sigma2 gamma) was set to G (1, 4.5), as recommended by Tong et al. (2015). The root age of the tree was set to 290.1 million years (the origin of Ensifera based on *Raphogla rubra*). The first 100,000 cycles were discarded as burn-in before we drew samples every 10 cycles over 500,000 cycles. Two independent runs produced stable and similar results in all analyses.

TVBOT 2.5.3 (Xie et al., 2023) was used to visualize the results of the divergence time trees generated by MCMCTree (see Supplementary Figure S11). The geologic time scale was based on the international chronostratigraphic chart v2023/06 (https://stratigraphy.org/chart#latest-version).

## 3 Results

### 3.1 Mitochondrial genome analysis

**FIGURE 1:**
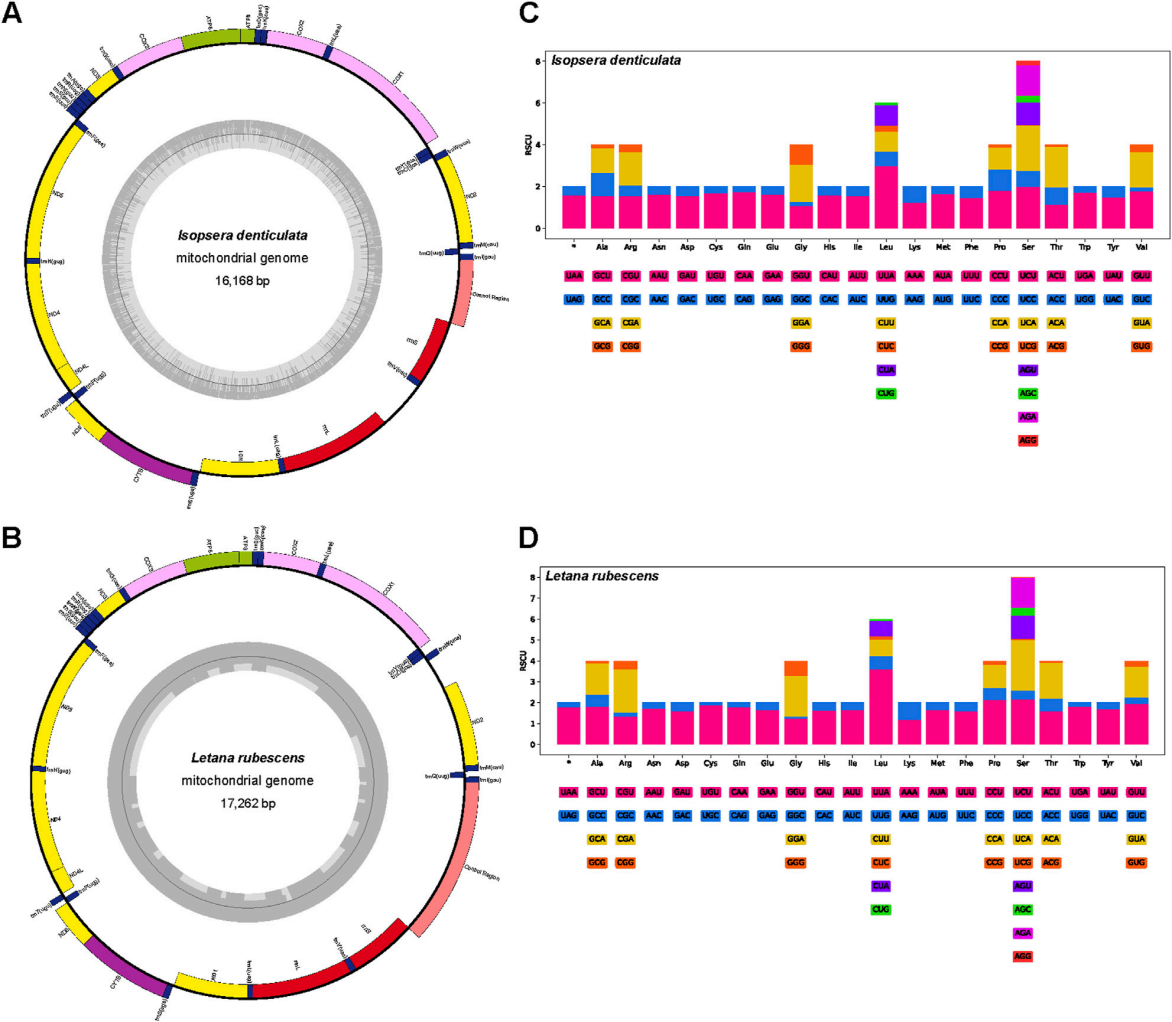
Structural map and relative synonymous codon usage (RSCU) of two mitochondrial genomes. The outer circle is encoded by the majority strand (+), and the second circle is encoded by the minority strand (−). Codon families (in alphabetical order) are provided below the horizontal axis. **(A)** Mitogenome map of *Isopsera denticulata*. **(B)** Mitogenome map of *Letana rubescens*. **(C)** RSCU of *Isopsera denticulata*. **(D)** RSCU of *Letana rubescens*.

Complete mitogenomes of *I. denticulata* (16,168 bp) and *L. rubescens* (17,262 bp) were sequenced and annotated. The mitogenomes of these species shared the same gene arrangement for 37 genes, of which 23 genes were located on the forward strand and others on the reverse strand (Supplementary Table S1 for *I. denticulata*; Supplementary Table S2 for *L. rubescens*). Both species maintained the most common gene order of the family Tettigoniidae (as shown in Figures 1A, B). Both newly sequenced mitogenomes were found to have similar nucleotide compositions, unveiling the typical AT-biased composition in Tettigoniidae and other insects (Guo et al., 2023; Huang et al., 2023). The AT content of the whole genome was 71.24% and 69.40% in *L. rubescens* and *I. denticulata*, respectively (Supplementary Table S3).

### 3.2 Protein-coding gene analysis

Among the 13 PCGs, the length of *ATP8* was the shortest, while that of *ND5* was the longest. In the *I. denticulata* mitogenome, except for the gene *ATP6* initiated with GTG, all PCGs used ATN as the start codon (Supplementary Table S1). However, except for the gene *ND1* started at TTG, all PCGs from *L. rubescens* used ATN as the start codon (Supplementary Table S2). The AT content of all PCGs in *I. denticulata* ranged from 63.7% to 72.8%, with an AT skew ranging from 0.015 to −0.268 (Supplementary Table S3). In *L. rubescens*, the AT content of each PCG ranged from 65.2% to 76.5%, with an AT skew ranging from −0.022 to −0.264 (Supplementary Table S3).

Relative synonymous codon usage (RSCU) analysis of *I. denticulata* and *L. rubescens* showed that a total of 64 codons were used (Figures 1C, D). The codon TTA (Leu) is the most frequently used codon in PCGs, appearing 313 times with an RSCU value of 2.98 in *I. denticulata* and 376 times with an RSCU value of 3.61 in *L. rubescens* (Supplementary Table S4).

### 3.3 Selective pressures and mean pairwise identity of Tettigoniidae

**FIGURE 2:**
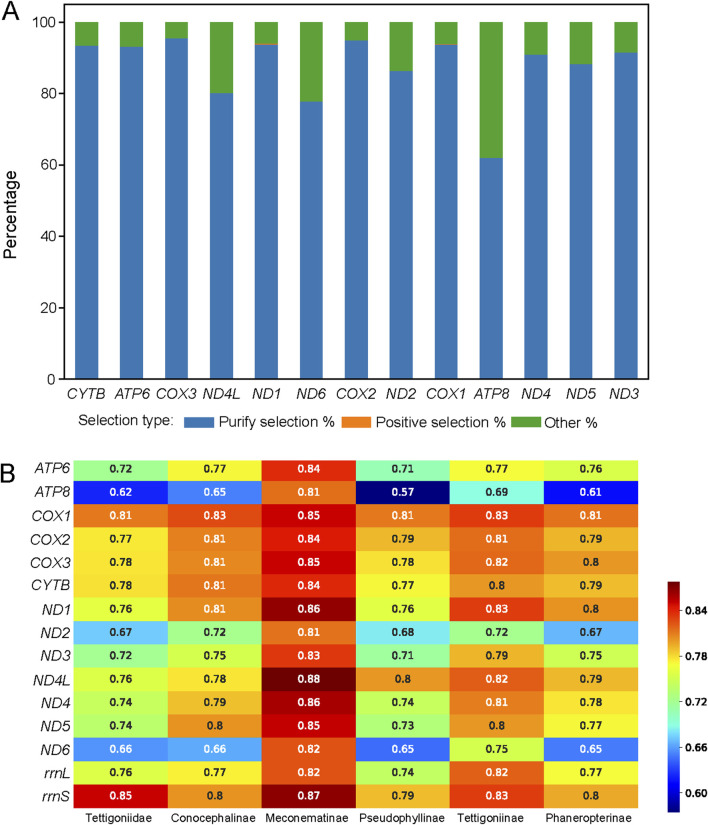
Selective pressure on sites of the 13 protein-coding genes across 111 species and mean pairwise identity for the five major subfamilies within Tettigoniidae. **(A)** Distribution of selective pressures on sites, as shown by a histogram. Sites under purifying selection are represented in blue, those under positive selection are represented in orange, and sites classified as “other” are represented in green. **(B)** The mean pairwise identity represents the evolutionary rate. The higher values indicate greater conservation (red), while lower values reflect greater divergence (blue).

The dN/dS ratio is a measure of the selective pressure acting on a gene, indicating neutral selection (dN/dS = 1), negative or purifying selection (dN/dS < 1), and positive or diversifying selection (dN/dS > 1). Among the analyzed genes, *COX3* exhibited the highest proportion of purifying selection, indicating strong evolutionary conservation, as shown in Figure 2A. In contrast, *ATP8* showed the lowest proportion of purifying selection, suggesting a lower selective pressure. Notably, only two genes, *ND1* and *COX1*, contained sites under positive selection, highlighting the specific areas of adaptive evolution.

In Tettigoniidae, the average evolutionary rate across 15 genes was 0.74. Among the subfamilies, Meconematinae had the highest mean evolutionary rate of 15 genes at 0.84, making it the slowest-evolving subfamily. The evolutionary rates of the 13 PCGs and 2 rRNAs in Meconematinae were found to be relatively high, suggesting that the mitochondrial evolutionary rate in this lineage is relatively conserved. Pseudophyllinae, with the lowest evolutionary rate of 0.73, was found to be characterized by pronounced mitochondrial differentiation and a high degree of diversification (Figure 2B). Among the five subfamilies, the average evolutionary rate of *ATP8* was 0.66, making it the most rapidly evolving gene, while *COX1* had the highest average evolutionary rate of 0.82, suggesting that it is the most conserved gene.

### 3.4 Alignment and tree evaluation

The information collected for the three datasets, such as the Akaike Information Criterion (AIC), BIC, saturation, SNR, and bootstrap of each node, is summarized in Table 1. All trees for each dataset are found in Supplementary Figures 1–9. According to the BIC, the optimal tree of each dataset was determined using the MP scheme. In terms of saturation, the tree of the AA dataset with the MP scheme had the lowest saturation, as shown in Table 1. For the SNR, P12R had the highest SNR, as shown in Table 1. Among the optimal trees for each dataset, the AA dataset had the highest number of reliable nodes (BS ≥ 95) with 91 nodes, while the P12R dataset had the fewest with 82 nodes.

### 3.5 The conflict node hypothesis

For the optimal trees of each dataset, we proposed four hypotheses to investigate the reasons for the key unreliable nodes. We used likelihood mapping to analyze the phylogenetic information contained in these nodes. Likelihood mapping analysis primarily targets nodes with a weak phylogenetic signal, such as hypotheses 1–3. Previous research has suggested that Mecopodinae and Pseudophyllinae form a sister group (Gaugel et al., 2023; Mugleston et al., 2013). However, in the phylogenetic relationships derived from mitogenomes, the affinities among Phaneropterinae, Mecopodinae, and Pseudophyllinae remain unclear.

**FIGURE 3:**
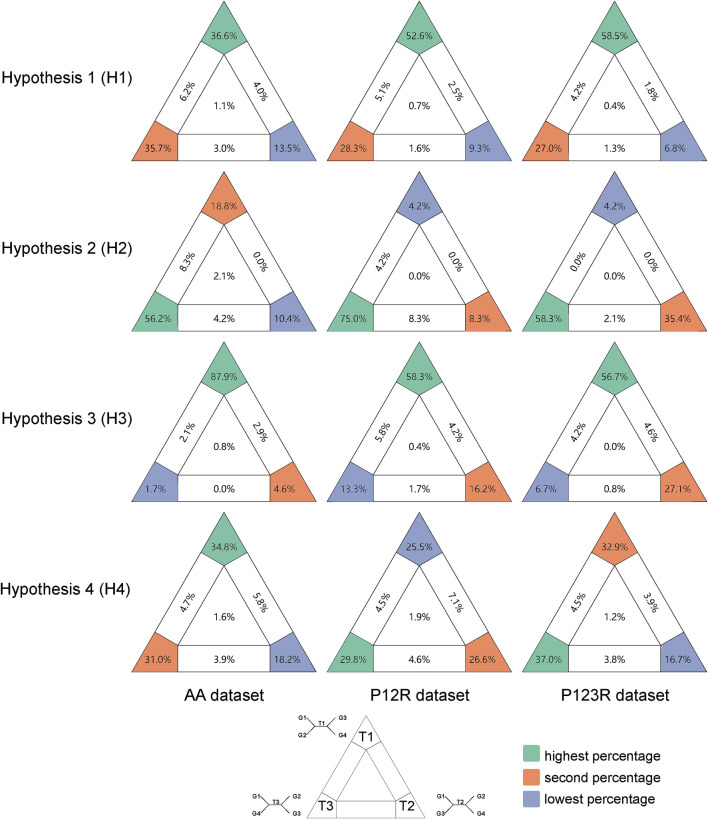
Results of likelihood mapping for the four hypotheses based on three datasets. Quartets falling in the three corners are deemed informative and categorized as fully resolved quartets. Those within the three rectangles are classified as partially resolved quartets, while those in the center are labeled as unresolved quartets. Among the fully resolved quartets, those with the highest percentage are labeled in green, the second in orange, and the lowest in blue-gray. The predefined groupings for T1–3 in each hypothesis are detailed in Materials and methods.

Among Anostostomatidae, Stenopelmatidae, Prophalangopsidae, Rhaphidophoridae, and Tettigoniidae, no stable and reliable phylogenetic relationship was obtained. Therefore, likelihood mapping was performed for hypotheses 1–3. For Hypothesis 1 in Figure 3, more than half of the signals in the P12R and P123R datasets supported Rhaphidophoridae as the sister group to the clade comprising Anostostomatidae, Stenopelmatidae, and Prophalangopsidae. In the AA dataset, the signals supporting this result were found to be slightly higher than those supporting the sister-group relationship of Anostostomatidae, Stenopelmatidae, and Prophalangopsidae with Tettigoniidae. All datasets supported the phylogenetic relationship of Anostostomatidae and Stenopelmatidae with Prophalangopsidae) (see Figures 3H2–3).

Hypothesis 4 aimed to determine whether the relationship between Mecopodinae and Phaneropterinae as sister groups is reliable, as shown in Figure 3. The AA dataset showed only weak support for a close relationship between Mecopodinae and Phaneropterinae, while ambiguous signals in the P123R and P12R datasets suggested a closer relationship between Pseudophyllinae and Phaneropterinae. All three datasets lacked clear phylogenetic signals regarding the relationships among Mecopodinae, Phaneropterinae, and Pseudophyllinae.

### 3.6 Phylogenetic relationships of Tettigoniidae

The Bayesian trees based on the AA matrix indicated that Prophalangopsidae is the sister group of Tettigoniidae, with strong support (PP = 0.98). In the ML trees, only the P12R matrix showed the closest phylogenetic relationship between Prophalangopsidae and Tettigoniidae. Both the P123R and AA matrices suggested that Tettigoniidae forms a sister group with a clade comprising Rhaphidophoridae, Prophalangopsidae, Stenopelmatidae, and Anostostomatidae. However, among all results, only the Bayesian tree employing the CAT-GTR model exhibited statistically high confidence.

Pseudophyllinae, Mecopodinae, and Phaneropterinae formed a clade, but there is not enough strong signal support for the relationships among these three subfamilies in the ML tree, Bayesian tree, and likelihood mapping analyses. The sister-group relationship between Mecopodinae and Phaneropterinae was displayed in both the ML tree and the Bayesian tree. However, likelihood mapping analyses indicated that neither amino acid nor nucleotide data contain clear phylogenetic signals. Mecopodinae was found to contain only Mecopodini. Pseudophyllinae was shown to include four tribes, with neither Phyllomimini nor Cymatomerini forming a monophyletic group. Callimenellini and Phyllomimus appeared to be embedded within Cymatomerini. The majority of tribes within Phaneropterinae formed complete monophyletic groups, except for Isopserini, which was embedded within Holochlorini.

Listroscelidinae, Conocephalinae, and Lipotactinae also formed a clade, but the phylogenetic position of Lipotactinae remains unclear due to only one sample. The sister-group relationship between Listroscelidinae and Lipotactinae was only displayed in the P123R datasets. In Conocephalinae, the three tribes were in a state of confusion. *Euconocephalus* was inserted within *Ruspolia*, and *Palaeoagraecia* clustered with *Pseudorhynchus*. The Bayesian tree also showed similar results.

In the optimal tree from the AA dataset, the relationship of Meconematinae as a sister group to the clade containing Bradyporinae and Tettigoniinae was well-supported (BS = 98). However, this relationship did not receive reliable support in the P12R and P123R datasets (BS < 95). The Bayesian analysis supported a close sister-group relationship between Bradyporinae and Tettigoniinae but rejected the hypothesis of a recent common ancestry between this clade (Bradyporinae + Tettigoniinae) and Meconematinae. The clade comprising Bradyporinae and Tettigoniinae was found to form a larger clade with Listroscelidinae, Conocephalinae, and Lipotactinae. However, the Bayesian tree lacked sufficient statistical support at this node. In Tettigoniinae, *Atlanticus sinensis* and *Anabrus simplex* formed a sister group. Pholidopterini and Gampsocleidini also formed a sister group. Platycleidini failed to form a monophyletic group.

Overall, the optimal tree from the AA dataset aligned more closely with the hypotheses from likelihood mapping, exhibiting less saturation and more reliable nodes. Within Tettigoniidae, the ML tree with the MP scheme based on the AA matrix showed strong statistical support. Therefore, the AA dataset and the topology derived from the MP scheme will be used for subsequent analyses.

### 3.7 Divergence time estimation

Based on fossil calibrations, the initial divergences within the Tettigoniidae were predicted to have occurred during the early Paleogene period (approximately 60.86 Mya), with the five major subfamilies also diverging during this era (Figure 5). The divergence times estimated from mitochondrial data are slightly more recent than those inferred from transcriptome data analysis during the late Cretaceous. Specifically, Pseudophyllinae diverged at approximately 49.02 Mya, followed by Phaneropterinae at approximately 48.05 Mya. The divergence time of Conocephalinae was estimated to be 38.78 Mya, while Tettigoniinae and Meconematinae have diverged at approximately 30.49 and 29.33 Mya, respectively. Bradyporinae had a later divergence, occurring approximately at 17.97 Mya. Based on mitogenomic data, we have provided an initial framework for exploring the internal evolutionary history of Tettigoniidae.

## 4 Discussion

The two mitogenomes of *L. rubescens* and *I. denticulata* were obtained by using next-generation sequencing in this study. There are no significant differences in the sizes of tRNAs, PCGs, and rRNAs within each species when compared. Previous studies have shown that nucleotide composition can affect codon usage, and the distinct codon usage pattern can result in differences in biological function (Foroughmand-Araabi et al., 2015; Huang et al., 2025). In Tettigoniidae, a strong bias in nucleotide composition (using A and T) has been revealed, and this situation is also found in our RSCU analysis.

A comparison of all currently available Tettigoniidae mitogenomes revealed unique variations in the gene order but high conservation in nucleotide composition and codon usage (Gaugel et al., 2023). Two groups within the Tettigoniidae exhibit changes in the region between the genes *rrnS* and *tRNA-Tyr*. Species of the Pseudophyllinae maintain the gene order of *tRNA-Met*, *tRNA-Ile*, and *tRNA-Gln*. In contrast, species of *Holochlora* and *Sinochlora* have relocated the position of *tRNA-Gln* between *ND2* and *tRNA-Trp*. In Pseudophyllinae, mitochondrial rearrangements are a notable characteristic that could serve as a molecular marker for this group. However, the variations observed in *Holochlora* and *Sinochlora* do not group into a specific taxonomic category, indicating that these changes may have occurred independently. Based on the mean pairwise identity, it is evident that the 13 PCGs and 2 rRNA genes exhibit different patterns of variation across different subfamilies (in Figure 2B). The mean pairwise identity is a tool for understanding the evolution of mitochondria across different clades. The *ATP6*, *ATP8*, *ND2*, and *ND6* genes exhibit significant diversity across four clades, except for Meconematinae. The relatively high conservation of these genes in Meconematinae compared to that of other clades may be due to the fact that this clade is represented by only one genus. However, the analysis of selective pressure shows that almost all genes are predominantly under purifying selection, with ATP8 having the lowest proportion at 62.12%.

Recent phylogenetic studies have focused on especially sparse data concerning the relationships among subfamilies within Tettigoniidae (Song et al., 2020). Our findings are in close agreement with a previous study (Gaugel et al., 2023), offering a more comprehensive view of the phylogenetic structure within Tettigoniidae. Tettigoniidae was represented by only two samples in the phylogeny of Orthoptera constructed using transcriptome data (Song et al., 2020). Our analysis based on mitogenome data has provided some new insights into the relationships among subfamilies. Compared to previous studies (Gaugel et al., 2023), the species coverage in our study was more than doubled (from 44 to 93 species in Tettigoniidae). With the continuous expansion of the dataset, we have obtained more detailed relationships across various taxonomic levels, from families, subfamilies, and tribes to even genera and species. Based on the phylogenetic results, we believe that three nodes warrant further explanation. Weak support for a sister-group relationship between Mecopodinae and Pseudophyllinae suggests that the mitochondrial data at this node may be subject to random error due to noise and an insufficient signal. We believe that this is the reason for the topological structure conflicts among these three subfamilies in current mitochondrial research (Gaugel et al., 2023). The sister-group relationship between Listroscelidinae and Lipotactinae was only displayed in the P123R datasets, despite being supported in previous studies (Gaugel et al., 2023). We argue that this divergence node is due to insufficient sampling of taxonomic units, as the high support values for node relationships indicate that the phylogenetic signals are abundant. The sister-group relationship between Prophalangopsidae and Tettigoniidae was recovered under the CAT + GTR model, and this result is consistent with the phylogenomics (Song et al., 2020). Under the homogeneous model, the internal relationships within Tettigoniideae were similar to those obtained from existing MrBayes analyses (Gaugel et al., 2023). We suggest that the heterogeneity among family-level taxa within Tettigoniideae can be resolved using the CAT + GTR model in PhyloBayes. The observed nuclear–mitochondrial discordance may be attributed to mitochondrial heterogeneity.

**FIGURE 4:**
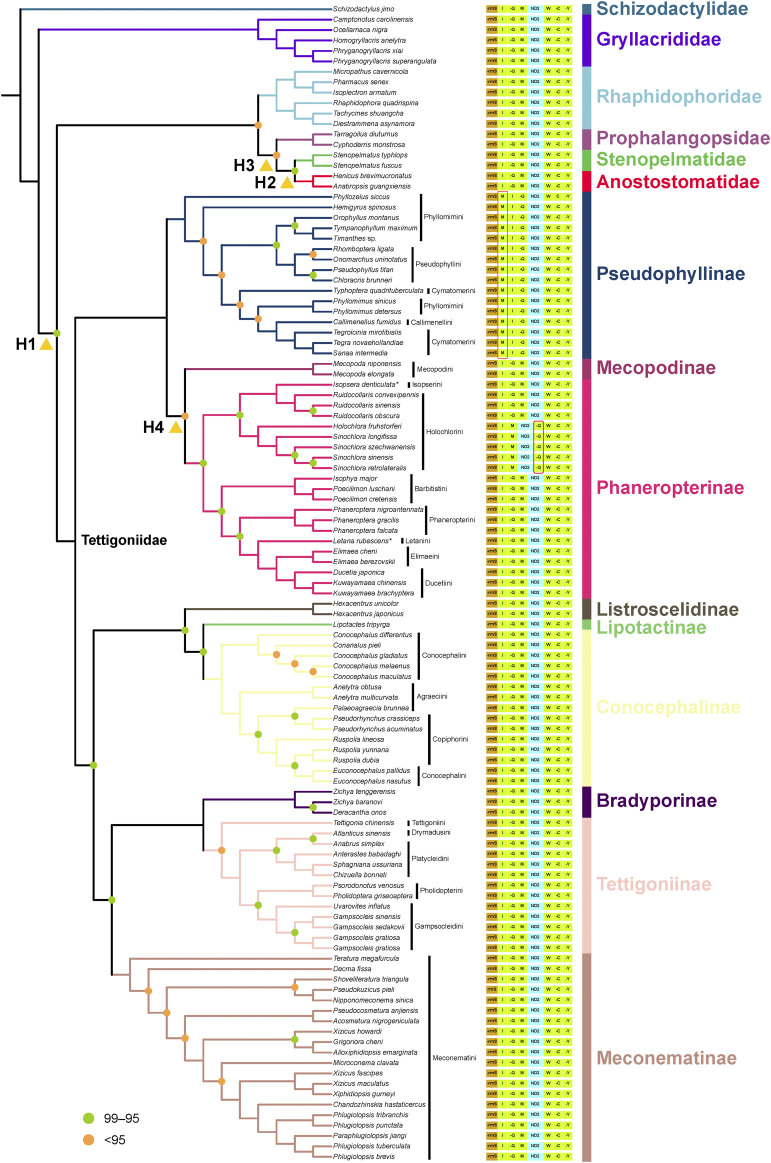
Phylogenetic relationships of Tettigoniidae are presented based on the optimal tree of the AA dataset. The species followed by an asterisk (*) are sequenced in this study. Nodes with 100 bootstrap support are not labeled. Nodes with support <95 are labeled in orange, and those with support between 95 and 99 are labeled in green. The figure displays a region of variable gene order in Tettigoniidae (from *rrnS* to *tRNA-Tyr*). The tRNA genes are denoted by their single-letter abbreviations, and gene names prefixed with “−” indicate those located on the negative strand. The yellow triangles mark the conflict nodes that were analyzed using likelihood mapping.

**FIGURE 5:**
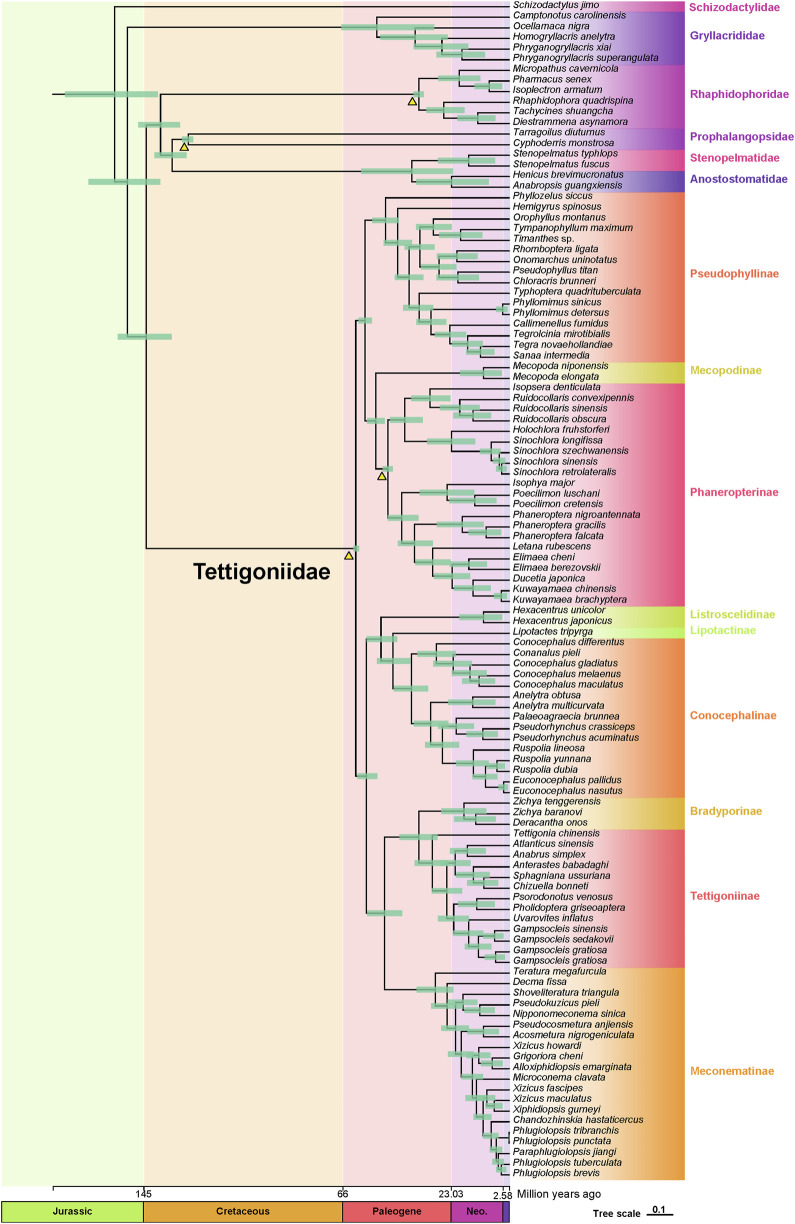
Dated phylogeny of Tettigoniidae based on the AA dataset and four fossil records. Green horizontal bars represent 95% credibility intervals. The fossil calibrations employed in this study are depicted as orange triangles. The scale axis of the tree is expressed in millions of years. The Quaternary is represented by purple rectangles, the Neogene is denoted by Neo., and other periods are not abbreviated.

We identified three major clades within this family, as shown in Figure 4: the first clade includes Pseudophyllinae, Mecopodinae, and Phaneropterinae; the second clade consists of Listroscelidinae, Lipotactinae, and Conocephalinae; and the third clade comprises Bradyporinae, Tettigoniinae, and Meconematinae. In our study, the latest mitochondrial dataset has further enriched our understanding of the tribes, genera, and species within Tettigoniidae. Our mitochondrial data strongly support the early Paleogene divergence of these three clades. Notably, the relatively recent divergence of Meconematinae corresponds to a greater degree of evolutionary conservatism (Figures 2B, 5). Further analysis of mitochondrial data saturation revealed that the rates of amino acid and nucleotide substitution were not high, indicating that mitochondrial amino acid sequences are valuable for assessing the phylogenetic relationships within Tettigoniidae (Gaugel et al., 2023). However, the likelihood mapping analysis revealed signal ambiguity at nodes H2 and H4, as shown in Figure 3, likely due to insufficient mitochondrial divergence or inadequate sample size.

Additional data in future analyses will be crucial to resolve these ambiguities. Furthermore, the recurrent tRNA positional shifts observed within the Tettigoniidae offer an intriguing area for future research, potentially shedding light on unique patterns of evolutionary dynamics and functional changes within this insect family.

## Data Availability

The data presented in the study are deposited in the NCBI GenBank repository, accession numbers PQ218338 and PQ218339.
