# Supplementary material for: Evolutionary history and divergence times of Tettigoniidae (Orthoptera) inferred from mitochondrial phylogenomics
Source: Front Genet. 2025 Mar 13;16:1495754. doi: 10.3389/fgene.2025.1495754 (PMC11966489; doi:10.3389/fgene.2025.1495754)
Supplement: Supplementary file 1 [file DataSheet1.pdf]

Table S1 Annotations and gene organization of *Isopsera denticulata*.

**Gene**	**Type**	**Start**	**End**	**Length**	**Start Codon**	**Stop Codon**	**Anticodon**	**Strand**
tRNA-Ile	tRNA	1	66	66			gau	+
tRNA-Gln	tRNA	64	131	68			uug	-
tRNA-Met	tRNA	131	196	66			cau	+
ND2	CDS	197	1213	1017	ATA	TAA		+
tRNA-Trp	tRNA	1216	1281	66			uca	+
tRNA-Cys	tRNA	1274	1336	63			gca	-
tRNA-Tyr	tRNA	1339	1403	65			gua	-
COX1	CDS	1449	3002	1554	ATA	TAA		+
tRNA-Leu	tRNA	3005	3070	66			uaa	+
COX2	CDS	3071	3757	687	ATG	TAA		+
tRNA-Lys	tRNA	3766	3834	69			cuu	+
tRNA-Asp	tRNA	3835	3899	65			guc	+
ATP8	CDS	3900	4064	165	ATC	TAA		+
ATP6	CDS	4058	4732	675	GTG	TAA		+
COX3	CDS	4732	5518	787	ATG	T		+
tRNA-Gly	tRNA	5519	5584	66			ucc	+
ND3	CDS	5582	5938	357	ATA	TAA		+
tRNA-Ala	tRNA	5943	6006	64			ugc	+
tRNA-Arg	tRNA	6008	6072	65			ucg	+
tRNA-Asn	tRNA	6078	6141	64			guu	+
tRNA-Ser	tRNA	6142	6202	61			gcu	+
tRNA-Glu	tRNA	6203	6267	65			uuc	+
tRNA-Phe	tRNA	6269	6334	66			gaa	-
ND5	CDS	6334	8066	1733	ATT	TA		-
tRNA-His	tRNA	8067	8130	64			gug	-
ND4	CDS	8131	9469	1339	ATG	T		-
ND4L	CDS	9463	9756	294	ATG	TAA		-
tRNA-Thr	tRNA	9759	9820	62			ugu	+
tRNA-Pro	tRNA	9820	9884	65			ugg	-
ND6	CDS	9886	10404	519	ATA	TAA		+
CYTB	CDS	10398	11540	1143	ATT	TAG		+
tRNA-Ser	tRNA	11539	11607	69			uga	+
ND1	CDS	11624	12574	951	ATT	TAG		-
tRNA-Leu	tRNA	12578	12642	65			uag	-
rrnL	rRNA	12643	13970	1328				-
tRNA-Val	tRNA	14559	14625	67			uac	-
rrnS	rRNA	14625	15410	786				-

Table S2. Annotations and gene organization of *Letana rubescens*.

Gene	Type	Start	End	Length	Start Codon	Stop Codon	Anticodon	Strand
tRNA-Ile	tRNA	1	64	64			gau	+
tRNA-Gln	tRNA	62	130	69			uug	-
tRNA-Met	tRNA	139	206	68			cau	+
ND2	CDS	207	1229	1023	ATG	TAA		+
tRNA-Trp	tRNA	1630	1696	67			uca	+
tRNA-Cys	tRNA	1696	1759	64			gca	-
tRNA-Tyr	tRNA	1764	1830	67			gua	-
COX1	CDS	1823	3364	1542	ATT	TAA		+
tRNA-Leu	tRNA	3367	3432	66			uaa	+
COX2	CDS	3434	4124	691	ATG	T		+
tRNA-Lys	tRNA	4125	4193	69			cuu	+
tRNA-Asp	tRNA	4194	4258	65			guc	+
ATP8	CDS	4259	4420	162	ATA	TAA		+
ATP6	CDS	4414	5091	678	ATG	TAA		+
COX3	CDS	5091	5878	788	ATG	TA		+
tRNA-Gly	tRNA	5878	5941	64			ucc	+
ND3	CDS	5939	6295	357	ATA	TAG		+
tRNA-Ala	tRNA	6297	6360	64			ugc	+
tRNA-Arg	tRNA	6361	6424	64			ucg	+
tRNA-Asn	tRNA	6428	6492	65			guu	+
tRNA-Ser	tRNA	6492	6558	67			gcu	+
tRNA-Glu	tRNA	6559	6625	67			uuc	+
tRNA-Phe	tRNA	6624	6689	66			gaa	-
ND5	CDS	6690	8421	1732	ATT	T		-
tRNA-His	tRNA	8422	8484	63			gug	-
ND4	CDS	8485	9820	1336	ATG	T		-
ND4L	CDS	9814	10113	300	ATG	TAA		-
tRNA-Thr	tRNA	10122	10184	63			ugu	+
tRNA-Pro	tRNA	10184	10248	65			ugg	-
ND6	CDS	10250	10771	522	ATT	TAA		+
CYTB	CDS	10771	11910	1140	ATG	TAA		+
tRNA-Ser	tRNA	11910	11977	68			uga	+
ND1	CDS	11994	12944	951	TTG	TAA		-
tRNA-Leu	tRNA	12945	13008	64			uag	-
rrnL	rRNA	13009	14332	1324				-
tRNA-Val	tRNA	14314	14384	71			uac	-
rrnS	rRNA	14384	15238	855				-

Table S3 Base composition and skewness of *Isopsera denticulata and Letana rubescens.*

	**AT skew**		**AT content (%)**	
	I. denticulate	*L. rubescens*	*I. denticulate*	*L. rubescens*
ATP6	-0.098	-0.095	66.3	69.8
ATP8	-0.051	-0.048	71.5	76.5
COX1	-0.102	-0.12	63.7	65.7
COX2	0.015	-0.1	68.2	67.8
COX3	-0.115	-0.144	64.3	65.2
CYTB	-0.088	-0.094	65.8	67.8
ND1	-0.25	-0.248	72.8	72.1
ND2	-0.081	-0.144	70.7	74.0
ND3	-0.088	-0.127	66.9	70.6
ND4	-0.268	-0.264	70.5	72.6
ND4L	-0.252	-0.214	72.8	74.6
ND5	-0.236	-0.202	70.5	74.1
ND6	-0.124	-0.068	71.7	73.2
rrnL	-0.058	-0.072	72.6	75.2
rrnS	-0.031	-0.022	70.4	73.0

Table S4 The RSCU of *Isopsera denticulata and Letana rubescens*. The asterisk represents termination codon.

Isopsera denticulata								*Letana rubescens*							
AA	Codon	Count	RSCU	AA	Codon	Count	RSCU	AA	Codon	Count	RSCU	AA	Codon	Count	RSCU
Phe	TTT	227	1.47	Ile	ATT	249	1.57	Phe	TTT	250	1.59	Ile	ATT	249	1.65
Phe	TTC	83	0.54	Ile	ATC	68	0.43	Phe	TTC	65	0.41	Ile	ATC	53	0.35
Leu	TTA	313	2.98	Met	ATA	198	1.66	Leu	TTA	376	3.61	Met	ATA	181	1.67
Leu	TTG	75	0.71	Met	ATG	41	0.34	Leu	TTG	66	0.63	Met	ATG	36	0.33
Ser	TCT	86	2.00	Thr	ACT	57	1.15	Ser	TCT	90	2.16	Thr	ACT	82	1.59
Ser	TCC	33	0.77	Thr	ACC	41	0.82	Ser	TCC	18	0.43	Thr	ACC	32	0.62
Ser	TCA	93	2.16	Thr	ACA	97	1.95	Ser	TCA	100	2.40	Thr	ACA	89	1.72
Ser	TCG	2	0.05	Thr	ACG	4	0.08	Ser	TCG	3	0.07	Thr	ACG	4	0.08
Tyr	TAT	119	1.50	Asn	AAT	116	1.62	Tyr	TAT	138	1.68	Asn	AAT	130	1.72
Tyr	TAC	40	0.50	Asn	AAC	27	0.38	Tyr	TAC	26	0.32	Asn	AAC	21	0.28
*	TAA	8	1.60	Lys	AAA	44	1.24	*	TAA	8	1.78	Lys	AAA	48	1.20
*	TAG	2	0.40	Lys	AAG	27	0.76	*	TAG	1	0.22	Lys	AAG	32	0.80
Cys	TGT	37	1.68	Ser	AGT	46	1.07	Cys	TGT	44	1.87	Ser	AGT	47	1.13
Cys	TGC	7	0.32	Ser	AGC	14	0.33	Cys	TGC	3	0.13	Ser	AGC	15	0.36
Trp	TGA	91	1.72	Ser	AGA	62	1.44	Trp	TGA	99	1.83	Ser	AGA	60	1.44
Trp	TGG	15	0.28	Ser	AGG	8	0.19	Trp	TGG	9	0.17	Ser	AGG	0.5	0.01
Leu	CTT	102	0.97	Val	GTT	94	1.77	Leu	CTT	81	0.78	Val	GTT	113	1.97
Leu	CTC	30	0.29	Val	GTC	11	0.21	Leu	CTC	19	0.18	Val	GTC	18	0.31
Leu	CTA	101	0.96	Val	GTA	89	1.68	Leu	CTA	77	0.74	Val	GTA	83	1.44
Leu	CTG	10	0.10	Val	GTG	18	0.34	Leu	CTG	6	0.06	Val	GTG	16	0.28
Pro	CCT	68	1.83	Ala	GCT	75	1.56	Pro	CCT	77	2.14	Ala	GCT	91	1.81
Pro	CCC	38	1.02	Ala	GCC	53	1.10	Pro	CCC	21	0.58	Ala	GCC	29	0.58
Pro	CCA	39	1.05	Ala	GCA	57	1.19	Pro	CCA	40	1.11	Ala	GCA	76	1.51
Pro	CCG	4	0.11	Ala	GCG	7	0.15	Pro	CCG	6	0.17	Ala	GCG	5	0.10
His	CAT	65	1.59	Asp	GAT	53	1.56	His	CAT	65	1.63	Asp	GAT	56	1.60
His	CAC	17	0.42	Asp	GAC	15	0.44	His	CAC	15	0.38	Asp	GAC	14	0.40
Gln	CAA	70	1.75	Glu	GAA	72	1.62	Gln	CAA	71	1.80	Glu	GAA	74	1.66
Gln	CAG	10	0.25	Glu	GAG	17	0.38	Gln	CAG	8	0.20	Glu	GAG	15	0.34
Arg	CGT	22	1.57	Gly	GGT	65	1.09	Arg	CGT	20	1.33	Gly	GGT	72	1.26

Arg	CGC	7	0.50	Gly	GGC	11	0.19	Arg	CGC	3	0.20	Gly	GGC	4	0.07
Arg	CGA	22	1.57	Gly	GGA	105	1.77	Arg	CGA	31	2.07	Gly	GGA	112	1.97
Arg	CGG	5	0.36	Gly	GGG	57	0.96	Arg	CGG	6	0.40	Gly	GGG	40	0.70

Table S5 Detailed information on representatives included in the present phylogeny. The newly sequenced samples are indicated with an asterisk (*).

**Superfamily**	**Family**	**Subfamily**	**ScientificName**	**Accession**	**Length**
Schizodactyloidea	Schizodactylidae		*Schizodactylus jimo*	NC_068225.1	16,428
Hagloidea	Prophalangopsidae		*Tarragoilus diuturnus*	NC_021397.1	16,144
Hagloidea	Prophalangopsidae		*Cyphoderris monstrosa*	NC_028059.1	16,590
Rhaphidophoroidea	Rhaphidophoridae	Aemodogryllinae	*Diestrammena asynamora*	NC_033989.1	15,309
Rhaphidophoroidea	Rhaphidophoridae	Aemodogryllinae	*Tachycines shuangcha*	NC_068194.1	16,723
Rhaphidophoroidea	Rhaphidophoridae	Macropathinae	*Pharmacus senex*	OR551713.1	15,251
Rhaphidophoroidea	Rhaphidophoridae	Macropathinae	*Isoplectron armatum*	OR551714.1	15,326
Rhaphidophoroidea	Rhaphidophoridae	Macropathinae	*Micropathus cavernicola*	OR551715.1	14,945
Rhaphidophoroidea	Rhaphidophoridae	Rhaphidophorinae	*Rhaphidophora quadrispina*	NC_067624.1	15,892
Stenopelmatoidea	Anostostomatidae	Anabropsinae	*Anabropsis guangxiensis*	NC_068726.1	16,148
Stenopelmatoidea	Anostostomatidae	Anostostomatinae	*Henicus brevimucronatus*	NC_028063.1	15,140
Stenopelmatoidea	Gryllacrididae		*Camptonotus carolinensis*	NC_028060.1	15,211
Stenopelmatoidea	Gryllacrididae		*Phryganogryllacris xiai*	NC_033994.1	15,876
Stenopelmatoidea	Gryllacrididae		*Homogryllacris anelytra*	NC_033998.1	15,706
Stenopelmatoidea	Gryllacrididae		*Phryganogryllacris superangulata*	NC_069838.1	15,976
Stenopelmatoidea	Gryllacrididae		*Ocellarnaca nigra*	NC_069865.1	16,510
Stenopelmatoidea	Stenopelmatidae		*Stenopelmatus fuscus*	NC_028058.1	15,767
Stenopelmatoidea	Stenopelmatidae		*Stenopelmatus typhlops*	NC_072277.1	15,806
Tettigonioidea	Tettigoniidae	Bradyporinae	*Deracantha onos*	NC_011813.1	15,650
Tettigonioidea	Tettigoniidae	Bradyporinae	*Zichya baranovi*	NC_033984.1	15,645
Tettigonioidea	Tettigoniidae	Bradyporinae	*Zichya tenggerensis*	MT849271.1	15,641
Tettigonioidea	Tettigoniidae	Conocephalinae	*Anelytra multicurvata*	NC_065467.1	14,851
Tettigonioidea	Tettigoniidae	Conocephalinae	*Anelytra obtusa*	NC_065466.1	15,549
Tettigonioidea	Tettigoniidae	Conocephalinae	*Conanalus pieli*	NC_033987.1	15,820
Tettigonioidea	Tettigoniidae	Conocephalinae	*Conocephalus differentus*	MF347703.1	16,038
Tettigonioidea	Tettigoniidae	Conocephalinae	*Conocephalus gladiatus*	MT849265.1	15,864
Tettigonioidea	Tettigoniidae	Conocephalinae	*Conocephalus maculatus*	NC_045065.1	15,905
Tettigonioidea	Tettigoniidae	Conocephalinae	*Conocephalus melaenus*	KY407794.1	15,923
Tettigonioidea	Tettigoniidae	Conocephalinae	*Euconocephalus nasutus*	NC_053383.1	14,999
Tettigonioidea	Tettigoniidae	Conocephalinae	*Euconocephalus pallidus*	MW009066.1	15,888
Tettigonioidea	Tettigoniidae	Conocephalinae	*Palaeoagraecia brunnea*	NC_067510.1	15,984

Tettigonioidea	Tettigoniidae	Conocephalinae	*Pseudorhynchus acuminatus*	NC_033992.1	16,056
Tettigonioidea	Tettigoniidae	Conocephalinae	*Pseudorhynchus crassiceps*	NC_033990.1	15,865
Tettigonioidea	Tettigoniidae	Conocephalinae	*Ruspolia dubia*	NC_009876.1	14,971
Tettigonioidea	Tettigoniidae	Conocephalinae	*Ruspolia lineosa*	NC_033991.1	16,110
Tettigonioidea	Tettigoniidae	Conocephalinae	*Ruspolia yunnana*	MZ128147.1	15,794
Tettigonioidea	Tettigoniidae	Lipotactinae	*Lipotactes tripyrga*	NC_033996.1	15,949
Tettigonioidea	Tettigoniidae	Listroscelidinae	*Hexacentrus japonicus*	NC_033983.1	16,120
Tettigonioidea	Tettigoniidae	Listroscelidinae	*Hexacentrus unicolor*	NC_033999.1	15,752
Tettigonioidea	Tettigoniidae	Meconematinae	*Acosmetura nigrogeniculata*	NC_045212.1	16,271
Tettigonioidea	Tettigoniidae	Meconematinae	*Alloxiphidiopsis emarginata*	NC_065298.1	16,207
Tettigonioidea	Tettigoniidae	Meconematinae	*Chandozhinskia hastaticercus*	NC_068777.1	15,627
Tettigonioidea	Tettigoniidae	Meconematinae	*Decma fissa*	NC_033981.1	16,122
Tettigonioidea	Tettigoniidae	Meconematinae	*Grigoriora cheni*	NC_068776.1	17,043
Tettigonioidea	Tettigoniidae	Meconematinae	*Microconema clavata*	MT849272.1	15,858
Tettigonioidea	Tettigoniidae	Meconematinae	*Nipponomeconema sinica*	MK903580.1	14,813
Tettigonioidea	Tettigoniidae	Meconematinae	*Paraphlugiolopsis jiangi*	NC_068778.1	16,085
Tettigonioidea	Tettigoniidae	Meconematinae	*Phlugiolopsis brevis*	NC_068752.1	16,553
Tettigonioidea	Tettigoniidae	Meconematinae	*Phlugiolopsis punctata*	NC_068775.1	17,461
Tettigonioidea	Tettigoniidae	Meconematinae	*Phlugiolopsis tribranchis*	OM892709.1	17,461
Tettigonioidea	Tettigoniidae	Meconematinae	*Phlugiolopsis tuberculata*	NC_068779.1	16,525
Tettigonioidea	Tettigoniidae	Meconematinae	*Pseudocosmetura anjiensis*	NC_033853.1	16,044
Tettigonioidea	Tettigoniidae	Meconematinae	*Pseudokuzicus pieli*	NC_033982.1	16,077
Tettigonioidea	Tettigoniidae	Meconematinae	*Shoveliteratura triangula*	NC_048466.1	16,152
Tettigonioidea	Tettigoniidae	Meconematinae	*Teratura megafurcula*	NC_060464.1	16,633
Tettigonioidea	Tettigoniidae	Meconematinae	*Xiphidiopsis gurneyi*	NC_039981.1	16,225
Tettigonioidea	Tettigoniidae	Meconematinae	*Xizicus fascipes*	OM892714.1	16,653
Tettigonioidea	Tettigoniidae	Meconematinae	*Xizicus howardi*	KY458226.1	16,146
Tettigonioidea	Tettigoniidae	Meconematinae	*Xizicus maculatus*	NC_040974.1	16,358
Tettigonioidea	Tettigoniidae	Mecopodinae	*Mecopoda elongata*	NC_021380.1	15,284
Tettigonioidea	Tettigoniidae	Mecopodinae	*Mecopoda niponensis*	NC_021379.1	15,364
Tettigonioidea	Tettigoniidae	Phaneropterinae	*Ducetia japonica*	KY612457.1	16,281
Tettigonioidea	Tettigoniidae	Phaneropterinae	*Elimaea berezovskii*	MT849266.1	16,232
Tettigonioidea	Tettigoniidae	Phaneropterinae	*Elimaea cheni*	GU323362.1	15,831

Tettigonioidea	Tettigoniidae	Phaneropterinae	*Holochlora fruhstorferi*	NC_033993.1	15,899
Tettigonioidea	Tettigoniidae	Phaneropterinae	*Isophya major*	NC_042666.1	15,724
Tettigonioidea	Tettigoniidae	Phaneropterinae	*Kuwayamaea brachyptera*	KT345950.1	16,237
Tettigonioidea	Tettigoniidae	Phaneropterinae	*Kuwayamaea chinensis*	NC_033995.1	15,875
Tettigonioidea	Tettigoniidae	Phaneropterinae	*Phaneroptera falcata*	KY458227.1	15,672
Tettigonioidea	Tettigoniidae	Phaneropterinae	*Phaneroptera gracilis*	NC_034756.1	18,255
Tettigonioidea	Tettigoniidae	Phaneropterinae	*Phaneroptera nigroantennata*	NC_034757.1	16,832
Tettigonioidea	Tettigoniidae	Phaneropterinae	*Poecilimon cretensis*	NC_077642.1	15,545
Tettigonioidea	Tettigoniidae	Phaneropterinae	*Poecilimon luschani*	NC_042665.1	15,568
Tettigonioidea	Tettigoniidae	Phaneropterinae	*Ruidocollaris convexipennis*	NC_046548.1	16,437
Tettigonioidea	Tettigoniidae	Phaneropterinae	*Ruidocollaris obscura*	NC_028160.1	16,424
Tettigonioidea	Tettigoniidae	Phaneropterinae	*Ruidocollaris sinensis*	MT849268.1	16,416
Tettigonioidea	Tettigoniidae	Phaneropterinae	*Sinochlora longifissa*	NC_021424.1	18,133
Tettigonioidea	Tettigoniidae	Phaneropterinae	*Sinochlora retrolateralis*	KC467056.1	17,209
Tettigonioidea	Tettigoniidae	Phaneropterinae	*Sinochlora sinensis*	MK903598.1	17,033
Tettigonioidea	Tettigoniidae	Phaneropterinae	*Sinochlora szechwanensis*	NC_034994.1	18,051
Tettigonioidea	Tettigoniidae	Phaneropterinae	*Isopsera denticulata* *	PQ218338	16,168
Tettigonioidea	Tettigoniidae	Phaneropterinae	*Letana rubescens* *	PQ218339	17,262
Tettigonioidea	Tettigoniidae	Pseudophyllinae	*Callimenellus fumidus*	ON012844.1	16,793
Tettigonioidea	Tettigoniidae	Pseudophyllinae	*Chloracris brunneri*	OM994955.1	18,446
Tettigonioidea	Tettigoniidae	Pseudophyllinae	*Hemigyrus spinosus spinosus*	OM994954.1	15,707
Tettigonioidea	Tettigoniidae	Pseudophyllinae	*Onomarchus uninotatus*	OM994957.1	16,305
Tettigonioidea	Tettigoniidae	Pseudophyllinae	*Orophyllus montanus*	KX057714.1	17,015
Tettigonioidea	Tettigoniidae	Pseudophyllinae	*Phyllomimus detersus*	NC_028158.1	16,007
Tettigonioidea	Tettigoniidae	Pseudophyllinae	*Phyllomimus sinicus*	NC_033997.1	15,692
Tettigonioidea	Tettigoniidae	Pseudophyllinae	*Phyllozelus siccus siccus*	OM937880.1	17,060
Tettigonioidea	Tettigoniidae	Pseudophyllinae	*Pseudophyllus titan*	NC_034773.1	16,227
Tettigonioidea	Tettigoniidae	Pseudophyllinae	*Rhomboptera ligata*	OM994952.1	17,375
Tettigonioidea	Tettigoniidae	Pseudophyllinae	*Sanaa intermedia*	OM994956.1	17,556
Tettigonioidea	Tettigoniidae	Pseudophyllinae	*Tegra novaehollandiae viridinotata*	KX057715.1	16,376
Tettigonioidea	Tettigoniidae	Pseudophyllinae	*Tegrolcinia mirotibialis*	OM994953.1	18,777
Tettigonioidea	Tettigoniidae	Pseudophyllinae	*Timanthes* sp.	ON023591.1	16,381
Tettigonioidea	Tettigoniidae	Pseudophyllinae	*Tympanophyllum maximum*	NC_077562.1	16,930

Tettigonioidea	Tettigoniidae	Pseudophyllinae	*Typhoptera quadrituberculata*	OM988187.1	17,718
Tettigonioidea	Tettigoniidae	Tettigoniinae	*Anabrus simplex*	NC_009967.1	15,766
Tettigonioidea	Tettigoniidae	Tettigoniinae	*Anterastes babadaghi*	NC_046894.1	15,883
Tettigonioidea	Tettigoniidae	Tettigoniinae	*Atlanticus sinensis*	MK903557.2	15,812
Tettigonioidea	Tettigoniidae	Tettigoniinae	*Chizuella bonneti*	MH685924.1	16,273
Tettigonioidea	Tettigoniidae	Tettigoniinae	*Gampsocleis gratiosa*	NC_011200.1	15,929
Tettigonioidea	Tettigoniidae	Tettigoniinae	*Gampsocleis gratiosa gratiosa*	MW092767.1	15,719
Tettigonioidea	Tettigoniidae	Tettigoniinae	*Gampsocleis sedakovii*	MK903561.2	15,774
Tettigonioidea	Tettigoniidae	Tettigoniinae	*Gampsocleis sinensis*	MT849267.1	15,939
Tettigonioidea	Tettigoniidae	Tettigoniinae	*Pholidoptera griseoaptera*	MT872693.1	16,112
Tettigonioidea	Tettigoniidae	Tettigoniinae	*Psorodonotus venosus*	MK951778.1	15,844
Tettigonioidea	Tettigoniidae	Tettigoniinae	*Sphagniana ussuriana*	KY783907.1	15,858
Tettigonioidea	Tettigoniidae	Tettigoniinae	*Tettigonia chinensis*	KX057727.1	16,224
Tettigonioidea	Tettigoniidae	Tettigoniinae	*Uvarovites inflatus*	KP098593.1	15,956

Table S6 Fossil calibration information used in the present study.

**Tree Node**	**Fossil species**	**Age range (Mya)**
Raphidophoridae	*Protroglophilus tachycinoides*	38.0–33.9
Prophalangopsidae	*Pseudaboilus wealdensis*	130.0–125.45
Tettigoniidae	*Locusta groenlandica*	61.6–59.2
Phaneropterinae	*Arethaea solterae*	50.3–46.2

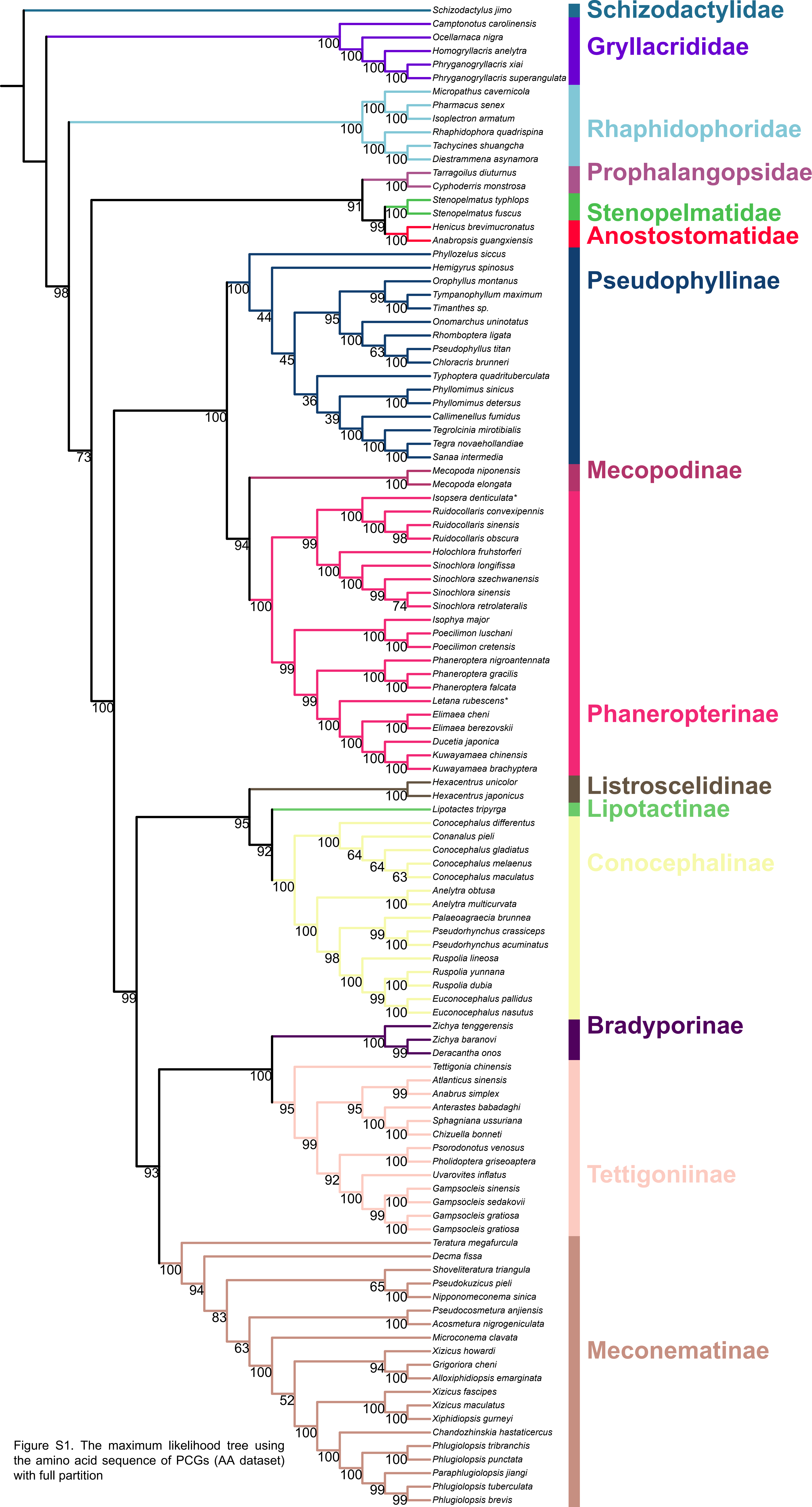

Figure S1. The maximum likelihood tree using the amino acid sequence of PCGs (AA dataset) with full partition

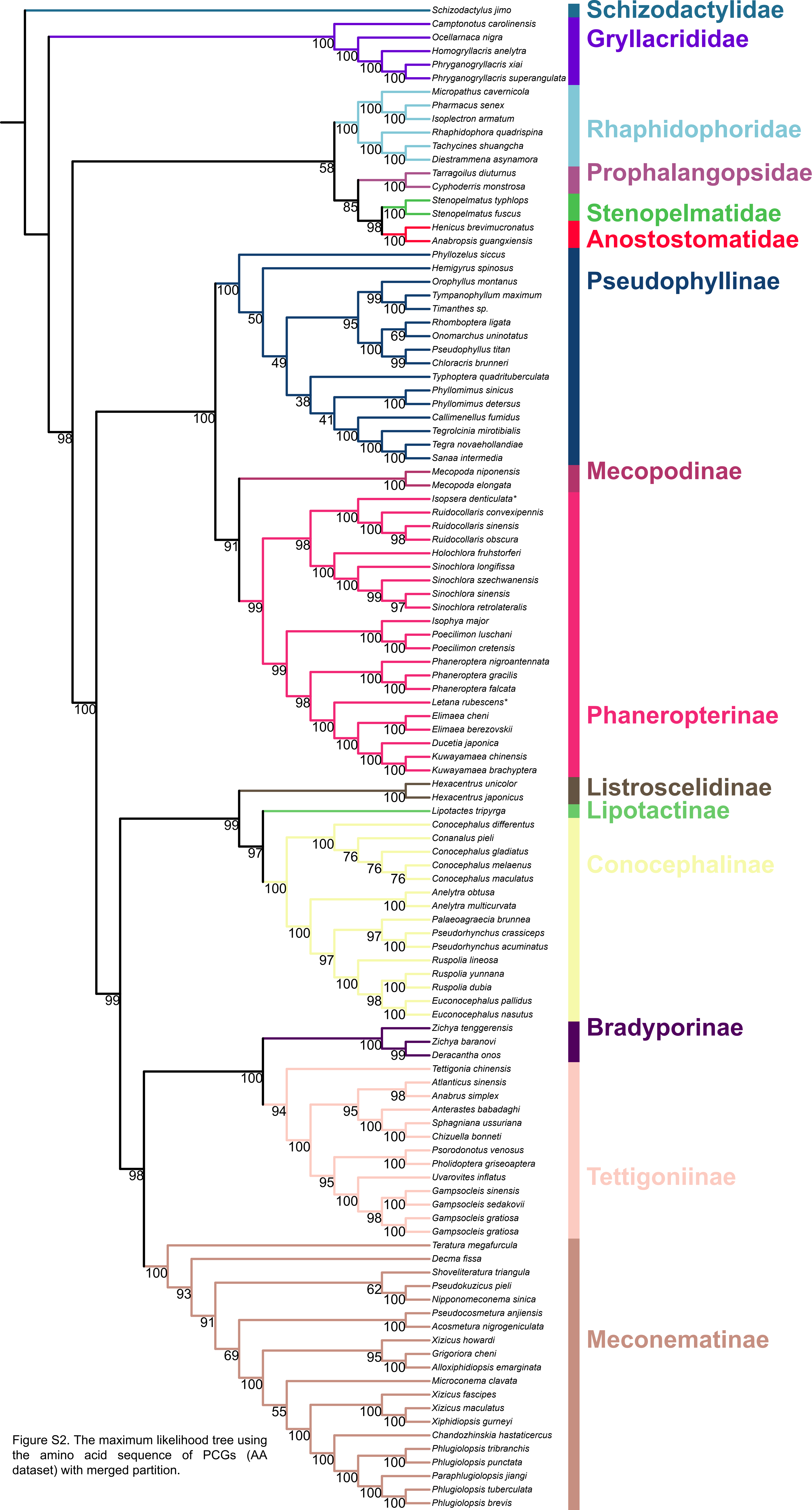

Figure S2. The maximum likelihood tree using the amino acid sequence of PCGs (AA dataset) with merged partition.

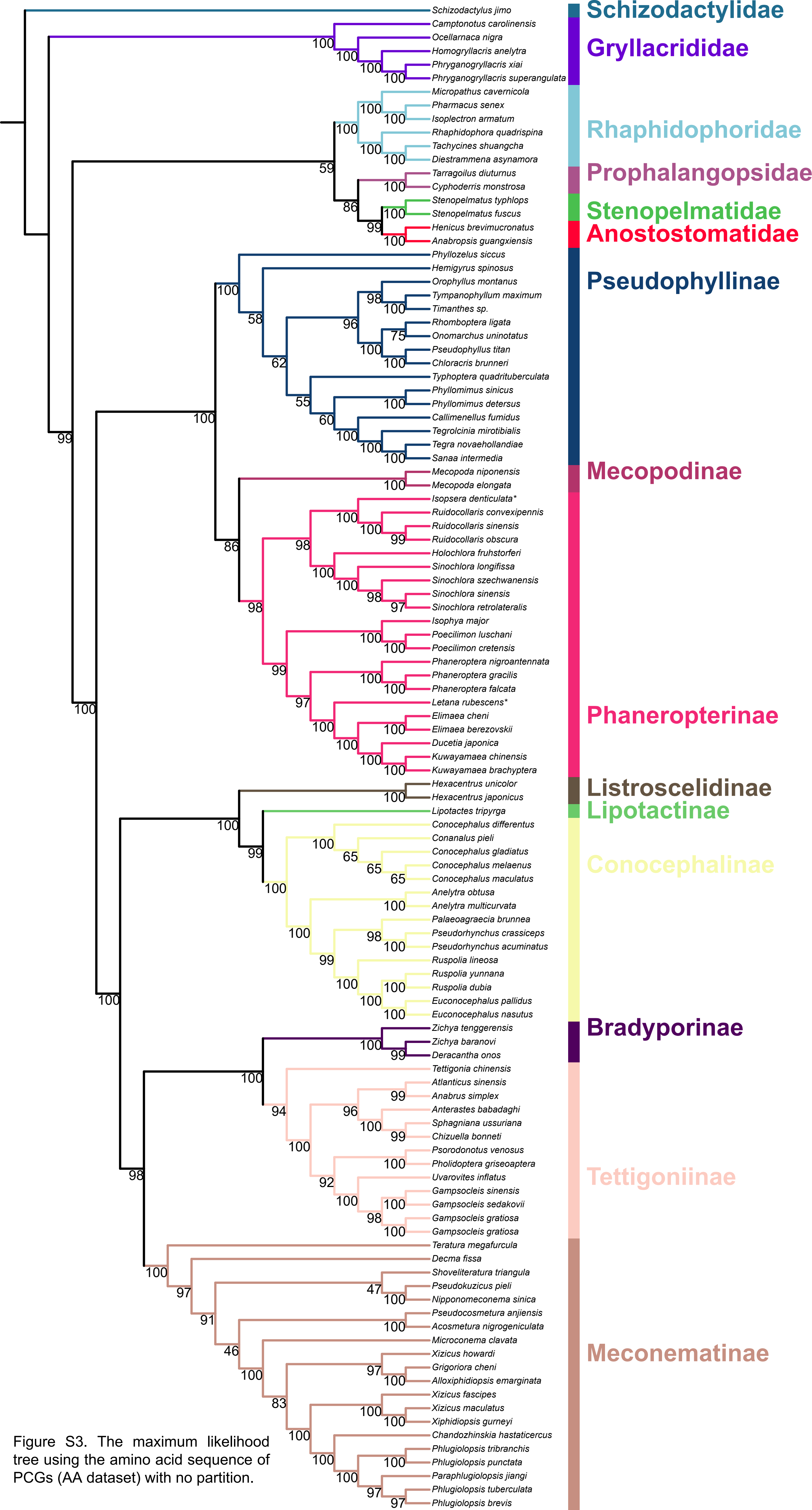

Figure S3. The maximum likelihood tree using the amino acid sequence of PCGs (AA dataset) with no partition.

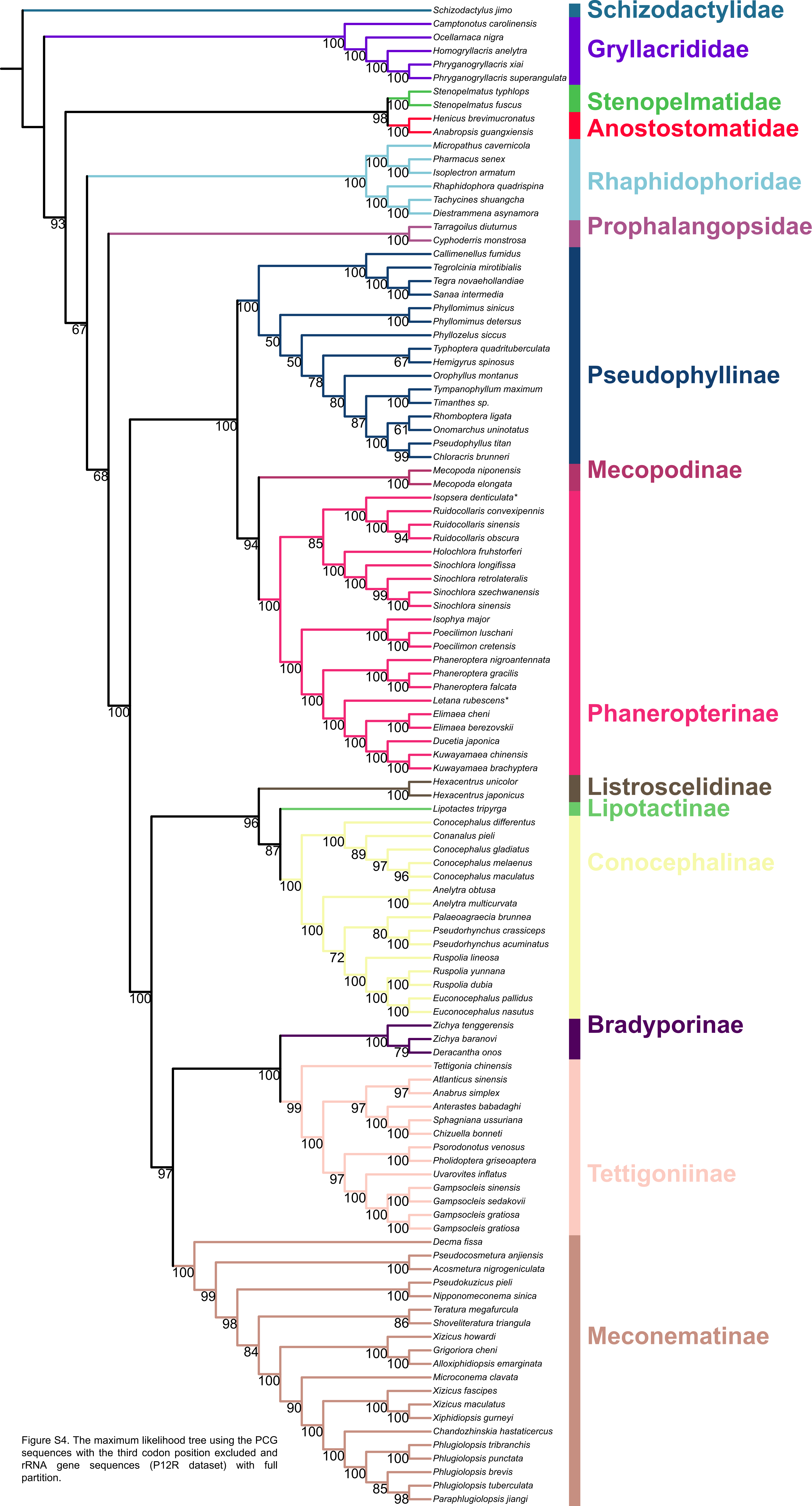

Figure S4. The maximum likelihood tree using the PCG sequences with the third codon position excluded and rRNA gene sequences (P12R dataset) with full partition.

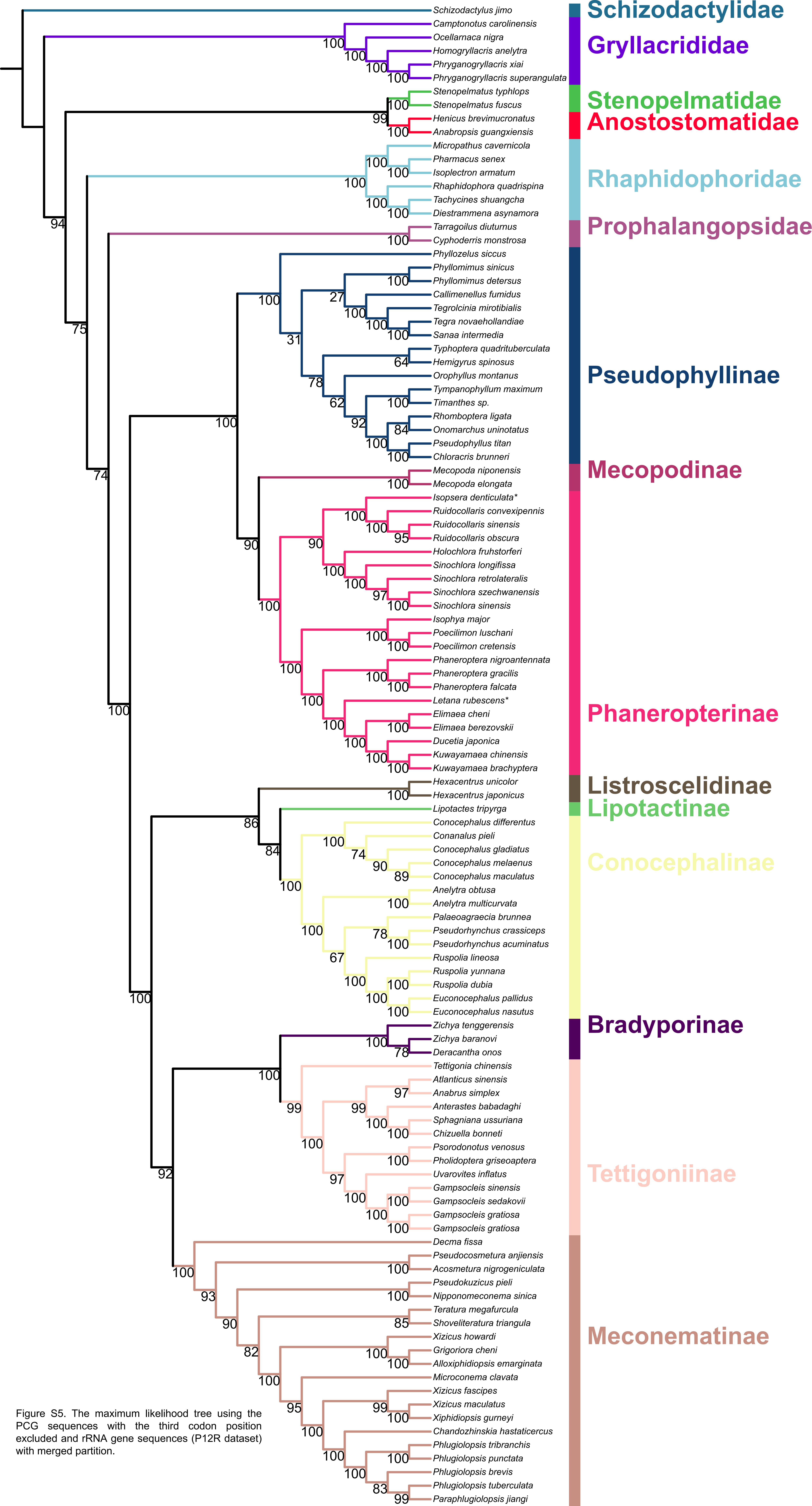

Figure S5. The maximum likelihood tree using the PCG sequences with the third codon position excluded and rRNA gene sequences (P12R dataset) with merged partition.

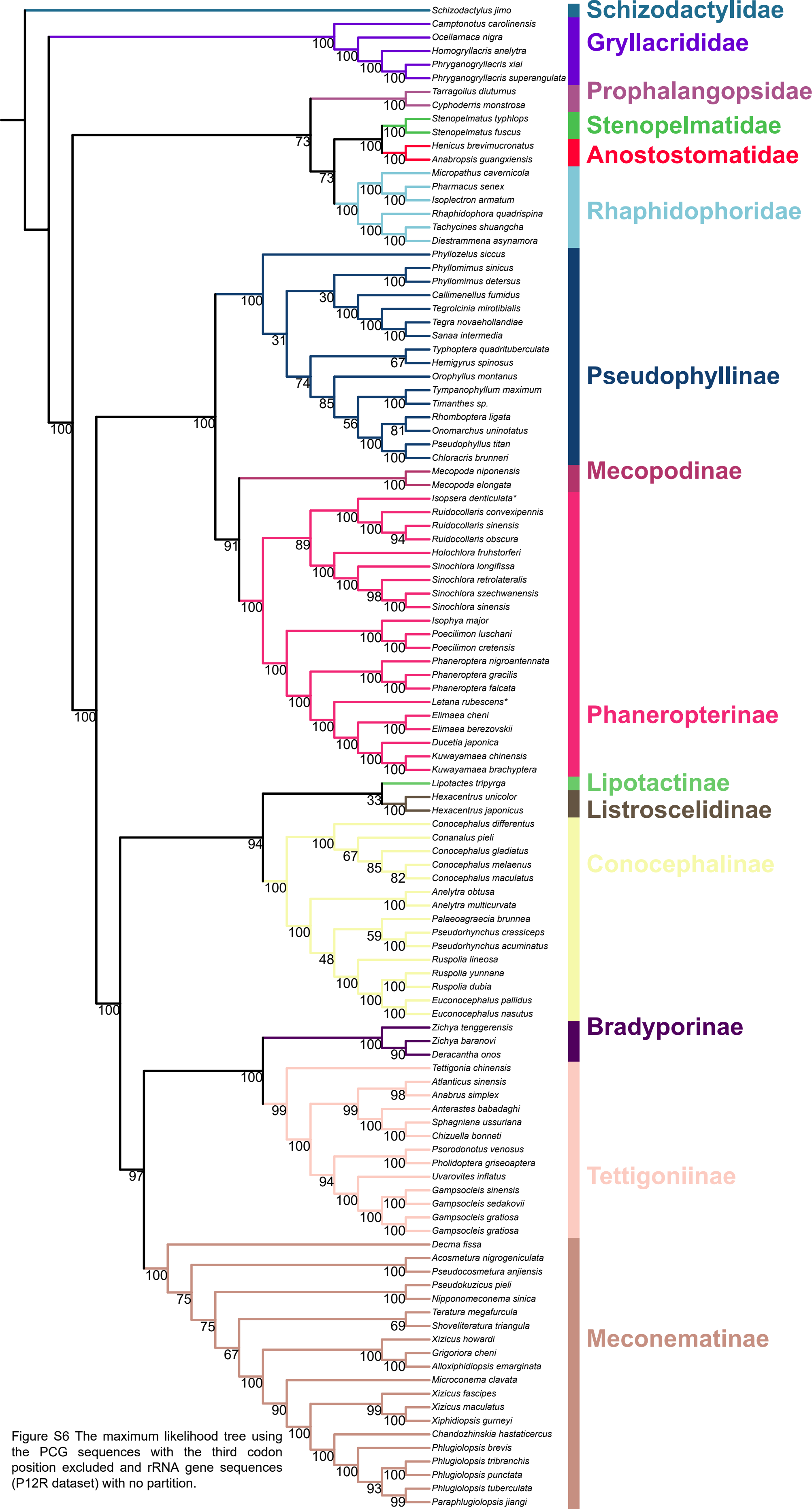

Figure S6 The maximum likelihood tree using the PCG sequences with the third codon position excluded and rRNA gene sequences (P12R dataset) with no partition.

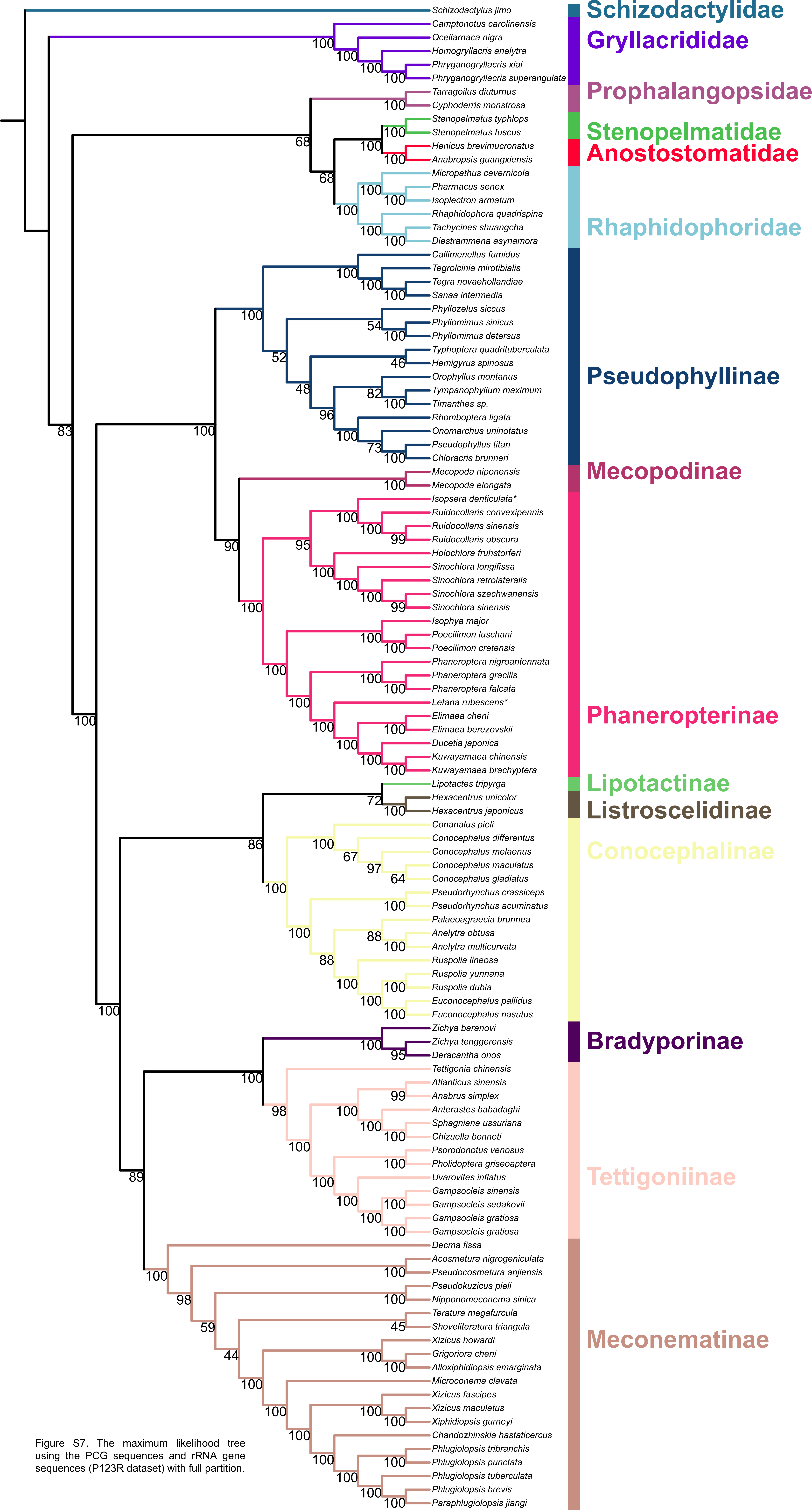

Figure S7. The maximum likelihood tree using the PCG sequences and rRNA gene sequences (P123R dataset) with full partition.

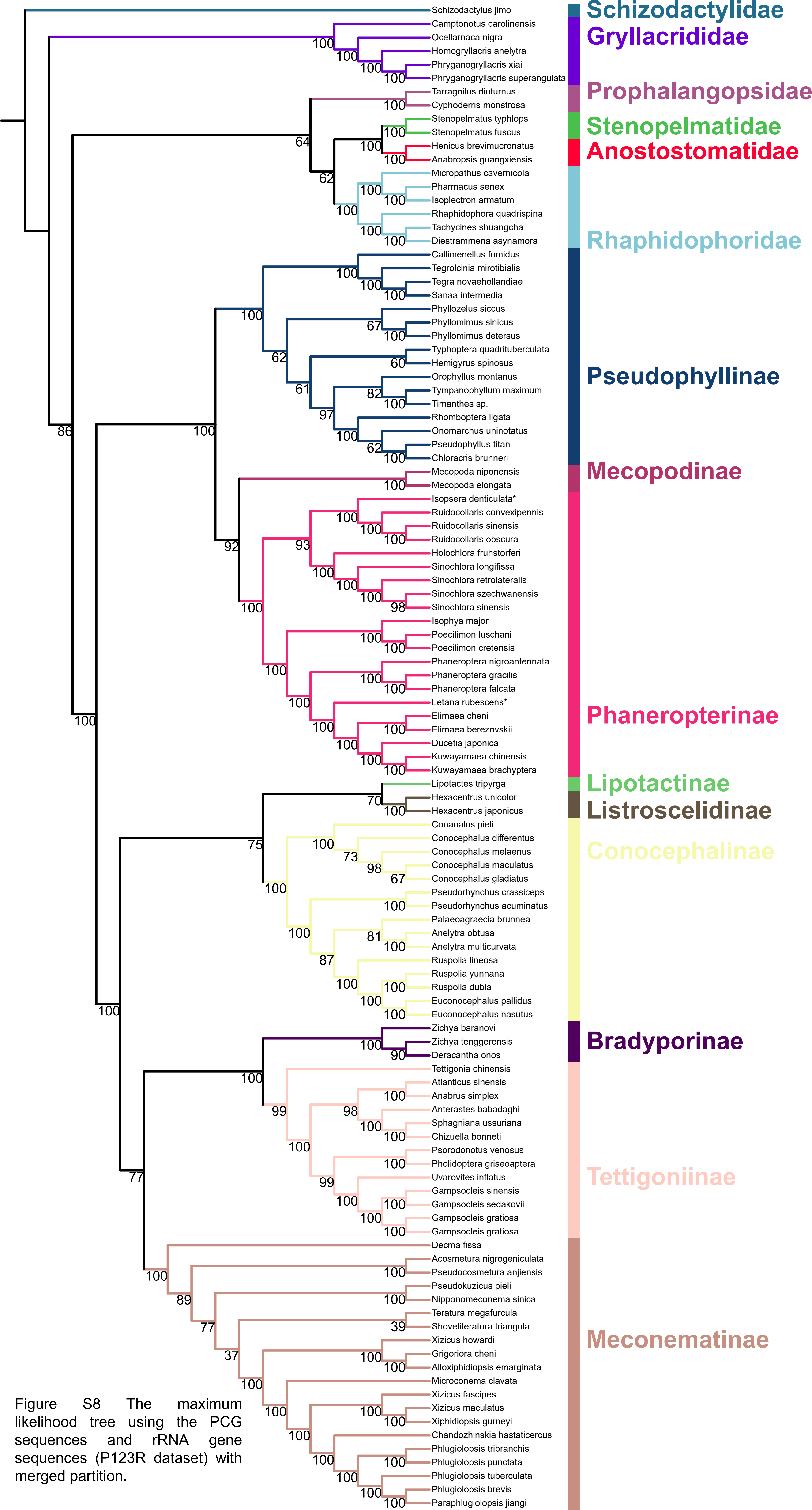

Figure S8 The maximum likelihood tree using the PCG sequences and rRNA gene sequences (P123R dataset) with merged partition.

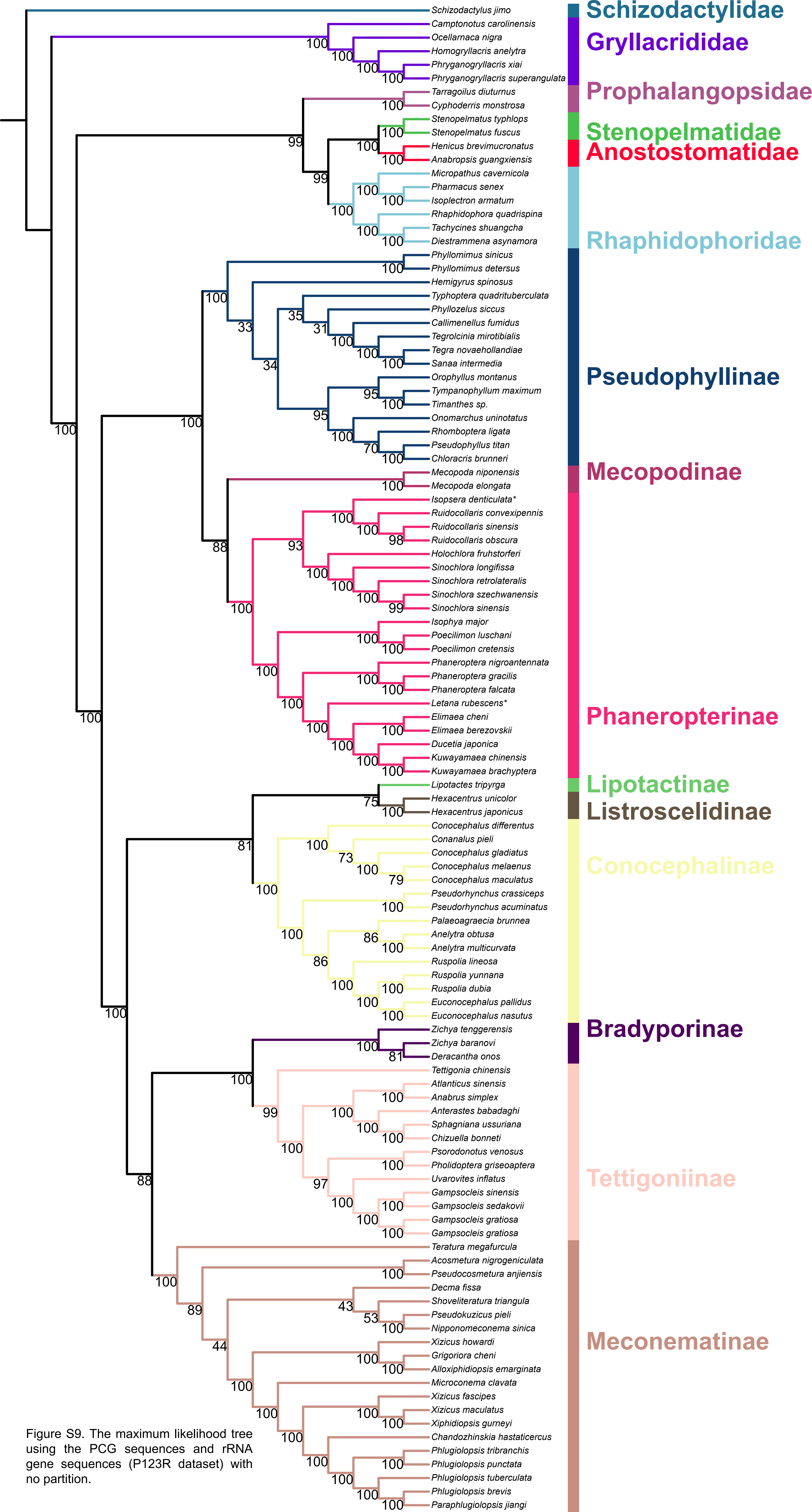

Figure S9. The maximum likelihood tree using the PCG sequences and rRNA gene sequences (P123R dataset) with no partition.

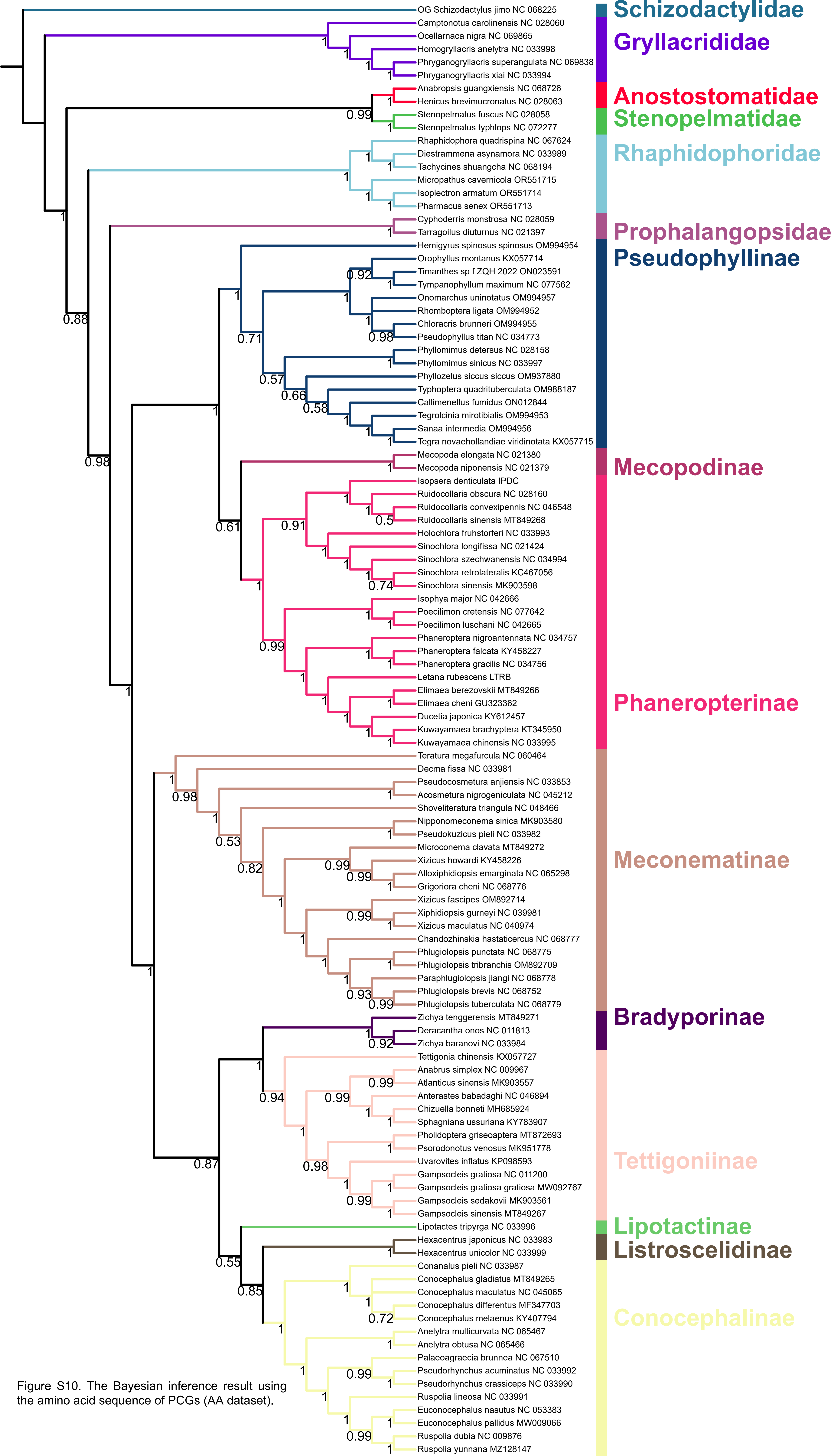

Figure S10. The Bayesian inference result using the amino acid sequence of PCGs (AA dataset).

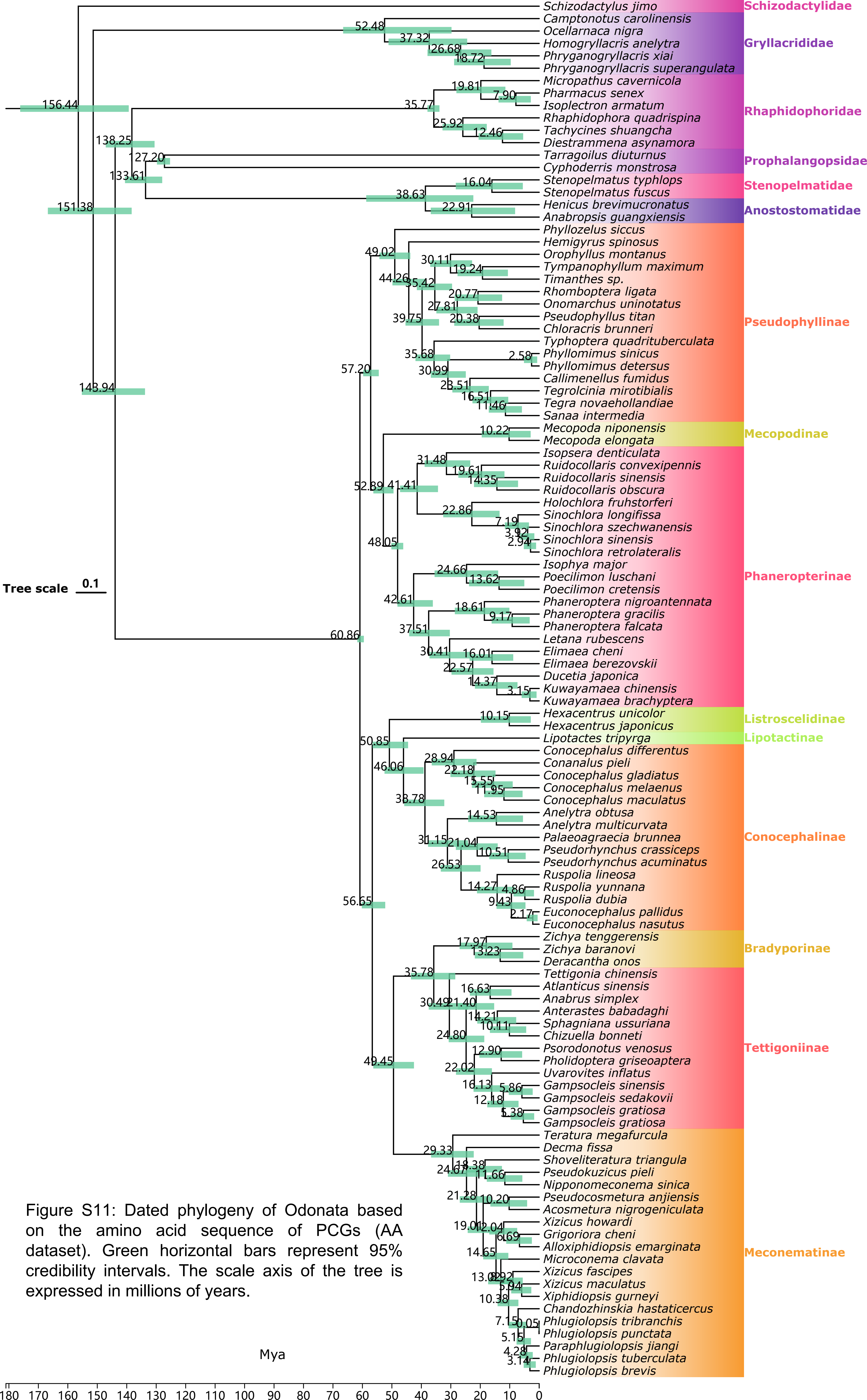

Figure S11: Dated phylogeny of Odonata based on the amino acid sequence of PCGs (AA dataset). Green horizontal bars represent 95% credibility intervals. The scale axis of the tree is expressed in millions of years.
